# Genomes of *Alteromonas australica,* a world apart

**DOI:** 10.1186/1471-2164-15-483

**Published:** 2014-06-18

**Authors:** Mario López-Pérez, Aitor Gonzaga, Elena P Ivanova, Francisco Rodriguez-Valera

**Affiliations:** División de Microbiología, Evolutionary Genomics Group, Universidad Miguel Hernández, Apartado 18, San Juan, 03550 Alicante Spain; Swinburne University of Technology, PO Box 218, Hawthorn, VIC, Melbourne 3122 Australia

**Keywords:** *Alteromonas australica*, Biogeography, *Alteromonas*, Genomic Island, Population genomics, Integron

## Abstract

**Background:**

*Alteromonas* is a genus of marine bacteria that is very easy to isolate and grow in the laboratory. There are genomes available of the species *Alteromonas macleodii* from different locations around the world and an *Alteromonas* sp. isolated from a sediment in Korea. We have analyzed the genomes of two strains classified by 16S rRNA (>99% similarity) as the recently described species *Alteromonas australica*, and isolated from opposite ends of the world; *A. australica* DE170 was isolated in the South Adriatic (Mediterranean) at 1000 m depth while *A. australica* H17^T^ was isolated from a sea water sample collected in St Kilda Beach, Tasman Sea.

**Results:**

Although these two strains belong to a clearly different species from *A. macleodii*, the overall synteny is well preserved and the flexible genomic islands seem to code for equivalent functions and be located at similar positions. Actually the genomes of all the *Alteromonas* species known to date seem to preserve synteny quite well with the only exception of the sediment isolate SN2. Among the specific metabolic features found for the *A. australica* isolates there is the degradation of xylan and production of cellulose as extracellular polymeric substance by DE170 or the potential ethanol/methanol degradation by H17^T^.

**Conclusions:**

The genomes of the two *A. australica* isolates are not more different than those of strains of *A. macleodii* isolated from the same sample. Actually the recruitment from metagenomes indicates that all the available genomes are found in most tropical-temperate marine samples analyzed and that they live in consortia of several species and multiple clones within each. Overall the hydrolytic activities of the *Alteromonas* genus as a whole are impressive and fit with its known capabilities to exploit sudden inputs of organic matter in their environment.

**Electronic supplementary material:**

The online version of this article (doi:10.1186/1471-2164-15-483) contains supplementary material, which is available to authorized users.

## Background

The genus *Alteromonas* contains species of marine bacteria that have been isolated often from the oceans around the world [1, 2]. The type species *Alteromonas macleodii* is found worldwide but mostly at temperate or tropical latitudes due to its mesophilic nature (grows between 10 and 45°C) (López-Pérez and Rodriguez-Valera, in press). This microbe was shown to be enriched in the particulate fraction of the water column in off-shore Mediterranean waters [3, 4]. In the Mediterranean Sea, bacteria of this species can be found even at high depths due to the relatively warm temperatures of its deep water mass (*ca*. 13°C) [5]. Related microbes have been found to bloom in mesocosms in the central Pacific gyre when the water was enriched with natural seawater nutrients [6, 7]. Other species of *Alteromonas* (*e.g*., *A. stellipolaris*) have been isolated from polar environments (Antarctic sea water) and are more psychrophilic [8].

Previously the genomes of two *A. macleodii* strains, AltDE and AltDE1 isolated from the South Adriatic at 1000 m depth were analyzed [9]. The average nucleotide identity (ANI) over the core genome between the two strains was 98.5%. From the same sample several other strains were isolated. The characterization by PCR amplification of their 16S rRNA gene classified one of them as belonging (99% identity) to the recently described species *Alteromonas australica* [10]. This bacterium (strain H17^T^) was collected from the first meters below the surface at St. Kilda beach, in Port Phillip Bay (Tasman Sea, Pacific Ocean). When comparing AltDE and AltDE1 [9] in spite of their high similarity, their genomes differ markedly in their content of flexible genomic regions or islands. These can be classified into two main types [11]. One type is typically associated to mobile genetic elements (including lysogenic phages). They tend to be found at the same genomic location and context but they are often completely absent from some strains. They have been designated "additive genomic islands" [11] because they typically vary by the presence/absence of gene cassettes in some strains but not in others. They carry lifestyle related genes, that include different metabolic properties (for example the degradation of organic compounds such as urea) or biotechnologically relevant features such as the polysaccharide or the polyketide synthase (PKS) clusters [12]. The second type are the "replacement genomic islands", in which completely different gene clusters with similar assigned functions are found at the same genomic location. They are typically involved in structural features of the cells exposed to the environment. Recent evidence indicates that they are exchanged by homologous recombination [13]. The gene cluster coding for the genes of the O-chain (sometimes called O-antigen) of the Gram negative lipopolysaccharide being a paradigmatic example.

This study aimed to provide a detailed genomic analysis of the two *A. australica* strains. We have fully sequenced and assembled these two genomes and compared them with all the available genomes of strains within the genus *Alteromonas*. The patterns of variation observed between the two *A. australica* strains are very similar to the ones found before when comparing the isolates from the same location mentioned above [9, 11]. Across the genus, the location and functional nature of the genomic islands was preserved, although obviously the genetic content was very different. However, some remarkable examples of conservation were found in the additive genomic islands with the members of *A. macleodii*.

## Results

**Figure 1:**
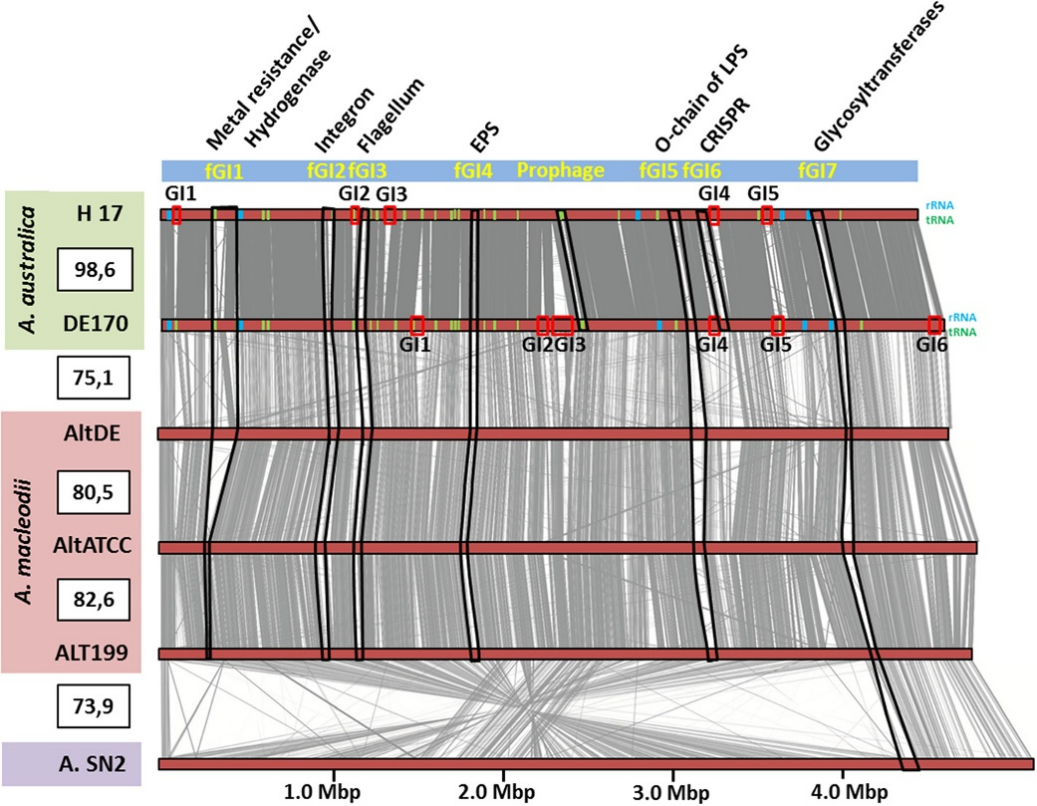
**Whole-genome alignment of** ***Alteromonas*** **genomes.** The genomes are arranged by decreasing ANI values. Colored rectangles to the left indicate members of the same species. fGIs identified in the comparison of both *A. australica* have been highlighted in black, identified by the inferred function on the top panel. Red rectangles indicate the location of the strains-specific genomic island. Blue and green vertical lines along the genomes of *A. australica* show the rRNA and tRNA locations respectively.

We have assembled into a single contig the genome of the two isolates, *A. australica* H17^T^ [10] isolated from the Tasman Sea and *A. australica* DE170 from the South Adriatic (16000 km away). The general features of the strains and the comparison with other members from the *Alteromonas* genus sequenced thus far are shown in Table 1. We have included in the comparison all previously assembled genomes of *Alteromonas* species [14–16] and the genome of the isolate *Alteromonas* sp. ALT199 from the Pacific Ocean, deposited as a draft genome (although it was assembled into one single contig) at the Joint Genome Institute (JGI, http://www.jgi.doe.gov/) (Table 1). In spite of the different locations and depth of the sampling sites, the two *A. australica* strains form a highly homogeneous clade with an average nucleotide identity (ANI) of 98.6%. The pairwise genome comparison of the *A. australica* isolates with all available strain genomes of the *Alteromonas* genus gave an ANI value of *ca*. 74% (Table 1). This is consistent with *A. australica* belonging to a different species within the same genus. The geographic origin of the isolates is presented in Additional file 1: Figure S1. Only 2224 genes were conserved in all the genomes shown in Table 1 (about 50% of the average genome gene content) (see Methods). Regarding the number of strain-specific genes, it was variable, ranging from 1170 for *Alteromonas* sp. SN2 to 260 for *A. australica* H17^T^ (Table 1). The alignment of the genomes showed that the synteny was remarkably well preserved in all the strains, except SN2, that showed large rearrangement from rRNA operon 1 to rRNA operon 5 (Figure 1).

**Table 1:** **General features of genomes**

***Alteromonas***strain	***A. australica***H17 ^T^	***A. australica***DE170	***A. macleodii***DE	***A. macleodii***ATCC 27126 ^T^	***Alteromonas***sp***.***ALT199	***Alteromonas***sp. SN2
Size (bp)	4308870	4457535	4480937	4607844	4635654	4972148
GC content (%)	44.9	44.9	44.9	44.6	43.7	43.5
Total ORFs	3764	4252	4346	4444	3955	4442
Share Genes	2289	2296	2260	2283	2279	2259
Unique Genes	260	780	523	542	561	1170
ANI (%)*	--	98.58	74.87	74.02	74.01	73.65
Origen isolated	Tasman Sea (Australia)	Adriatic Sea	Adriatic Sea	Pacific Ocean (Hawaii)	Pacific Ocean (San Diego)	Yellow Sea (South Korea)
Depth of Sample (m)	Surface	1000	1000	Surface	Surface	Surface

*ANI, Average nucleotide identity (Konstantinidis and Tiedje, 2005) to *A. australica* H17^T^ homologous genes.

**Figure 2:**
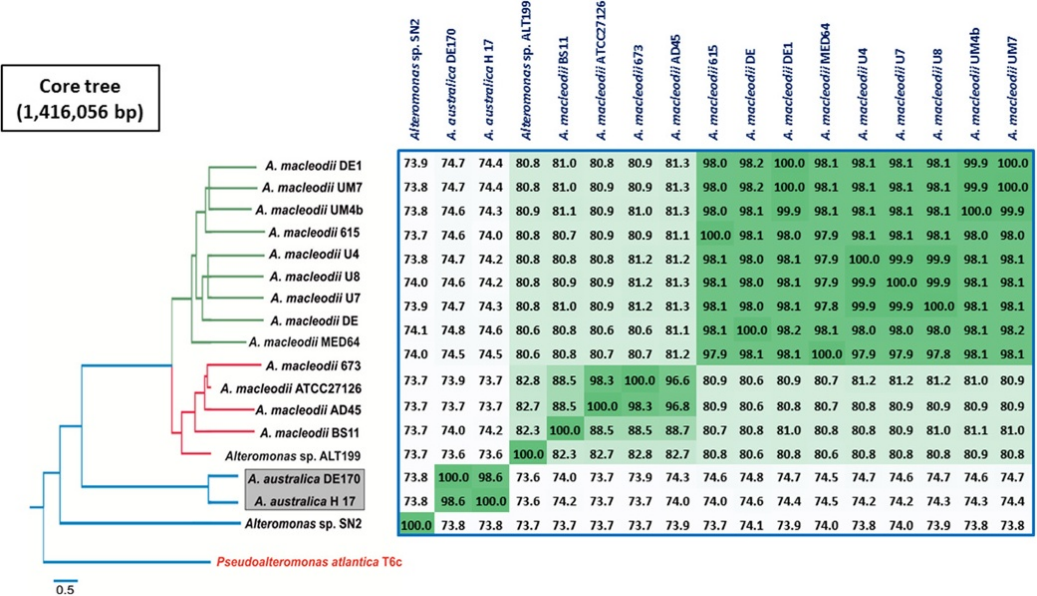
**Phylogenomic tree.** Phylogenetic tree constructed using a concatenated of the core proteome in all genomes of available *Alteromonas* genomes. *Pseudoalteromonas atlantica* T6c was used as outgroup. Table shows the ANI of the genomes pairwise comparison.

To investigate the phylogenetic relationships of both *A. australica* strains within the *Alteromonas* genus, whole-genome phylogeny was inferred from a concatenation of shared genes (*ca*. 1.4 Mb) including all the completely sequenced species (Figure 2). *Pseudoalteromonas atlantica* T6c, a marine bacterium which belong to the separate family *Pseudoalteromonadaceae* [17], was used as an outgroup. The *A. macleodii* isolates are split by the surface (small particle)/deep (larger particle) ecotypes (or subspecies) [11, 14, 18] while *A. australica* and *Alteromonas* sp. SN2 appear clearly separated as expected from different species of the same genus. *Alteromonas* sp. ALT199 clustered within the same branch of the members of the *A. macleodii* surface clade, formed by the strains AD45, BS11, 673 and the type strain ATCC 27126^T^ [18].

In order to analyzed the impact of the homologuos recombination in the evolution of the core genome of the members of the *Alteromonas* genomes we used ClonalFrame software v1.2 [19] to estimate two different values, *r/m* and *ρ/θ*. The parameter *r/m* shows the weight of recombination in the diversification of the species relative to mutation, whereas *ρ/θ* measures the absolute number of events of recombination relative to mutation. The results showed that within the *Alteromonas* genus the 95% credibility interval of *ρ/θ* was 0.01-0.02 (mean = 0.01), indicating that recombination happened hundred times less frequently than mutation. However, the impact of recombination had a predicted significant effect in the evolutionary process as showed by the *r/m* value of, 0.60 (with 95% credibility interval 0.45-0.91), suggesting that recombination introduces several polymorphisms per event. The average size of the fragments affected by recombination was 1102 bp (with a 95% credibility interval of 799 to 1349 bp), similar to those found in other bacteria, such as *Bacillus cereus* [20] and *Chlamydia psittaci* [21]. In a previous study carried out with the nine genomes belonging to the *A. macleodii* deep ecotype [11] we obtained similar results for the comparison of all the *A. macleodii* members (deep and surface ecotype) with values of *r/m* = 0.45 and *ρ/θ* = 0.06. However, when only the deep ecotype members were considered, both parameters were higher, *ρ/θ* (0.58) and *r/m* (5.13), suggesting that recombination is an important factor in the evolution within this clade. Using the locally collinear blocks generated from the whole-genome alignments we have also analyzed the existence of recombination events by building ML trees of randomly selected regions (Additional file 1: Figure S2). The results show frequent variations of the topology suggesting recombination events taking place across the *Alteromonas* genus, but only in small fragments of less than 3 Kb. However, topology was stable when fragments of more than 5 Kb were selected.

### Flexible genomic islands

The alignment of the two *A. australica* genomes and of other representative species of *Alteromonas* is shown in Figure 1. As has been shown several times [9, 11, 22–25] a significant part of the flexible genome is found in genomic islands larger than 15 Kb. As previously [11], we have classified them into two categories, the flexible genomic islands (fGIs) that are found in both isolates and at equivalent position in the genomes (same genome context) but contain different genes, and the unique islands that are found in either one or the other strain (Table 2). fGIs preserve the same location (with regard to the origin of replication) and the same genome context (neighboring genes) even when different species of the same genus are considered (Figure 1). This is remarkable since it shows that the location of these genomic features transcends the species and therefore, likely, very long evolutionary times. As found in *A. macleodii,* in *A. australica* there were two types of fGIs, the additive and the replacement types [11, 13].

**Table 2:** **Characteristics of genomic islands found in** ***A. australica*** **H17** ^**T**^ **and DE170 genomes**

Strain	No.	Start	End	Size (Kb)	No. genes	GC (%)	Inferred character
**Flexible genomic islands**							
H17-DE170	fGI-1	310522	384692	74	46	40.74	Metal resistance/Hydrogenases
H17-DE170	fGI-2	634953	650653	16	25	40.82	Integron
H17-DE170	fGI-3	1127948	1152459	25	25	37.52	Flagellum
H17-DE170	fGI-4	1783555	1790698	7	6	36.22	Exopolysaccharide
H17-DE170	fGI-5	3012274	3028621	16	16	38.40	O-chain of LPS
H17-DE170	fGI-6	3188385	3217385	29	29	42.80	CRISPR cluster
H17-DE170	fGI-7	3853337	3872202	19	16	38.35	Glycosyltransferases
**Strain specific genomic islands**							
H 17	GI-1	79701	110606	31	19	41.84	DNA phosphorothioation
H 17	GI-2	953402	975953	23	17	43.29	Type I RM system
H 17	GI-3	1298603	1317990	19	28	38.46	ND
H 17	GI-4	3133024	3168117	35	25	44.53	Carbohydrates metabolism
H 17	GI-5	3429815	3464787	35	26	42.53	PQQ dehydrogenase
DE170	GI-1	1444412	1484731	40	54	46.07	Mu Phage
DE170	GI-2	2167586	2188175	20	12	44.30	Type I RM system
DE170	GI-3	2246523	2282575	36	39	43.82	Metal resistance
DE170	GI-4	3139215	3155614	16	12	44.6	Xylan catabolism
DE170	GI-5	3713976	3741976	28	26	46.17	Cellulose synthase cluster
DE170	GI-5	4389242	4419286	30	33	42.57	MGI

Abbreviations: *LPS* Lipopolysaccharide, *CRISPR* clustered regularly interspaced short palindromic repeats, *RM* restriction modification, *ND* Not determined, *PQQ* pyrroloquinoline quinone, *MGI* Mobilizable genomic island.

### Additive fGIs

fGI1 is of the additive kind and is a typical example of genomic island in which a conserved tRNA act as an insertion target for gene cassetes [18]. Addittion of different cassettes makes the length variable among the strains, going from more than 110 Kb in *A. macleodii* DE (AltDE) and *A. australica* DE170 to a single cassette in *A. macleodii* ATCC 27126^T^ (Additional file 1: Figure S3). Two different kinds of cassettes were found, one is composed of different clusters of *czc*ABC genes, encoding the components of heavy metal efflux pumps important for resistance to cobalt, zinc and cadmium. The other contains a complete hydrogenase cluster. It is remarkable that this hydrogenase cluster previously described in the strain AltDE [14] from the Adriatic Sea, has been also found with a similarity higher than 99% in the Tasman Sea isolate H17^T^ (Additional file 1: Figure S3). However, it is not present in *A. australica* strain DE170 that comes from the same water sample than AltDE. This cluster is also present in all the members of the *A. macleodii* deep clade, with the single exception of the strain *A. macleodii* 615 isolated from the English Channel. The AltDE cluster has been expressed as an active enzyme in both *E. coli* [26] and in the cyanobacterium *S. elongates* [27]. A similar (87% nucleotide identity) hydrogenase cluster was found in a *Glaciecola* sp. 4H-3-7 + YE-5 (an isolate from marine subseafloor sediments Suruga Bay, Japan) plasmid [28].

**Figure 3:**
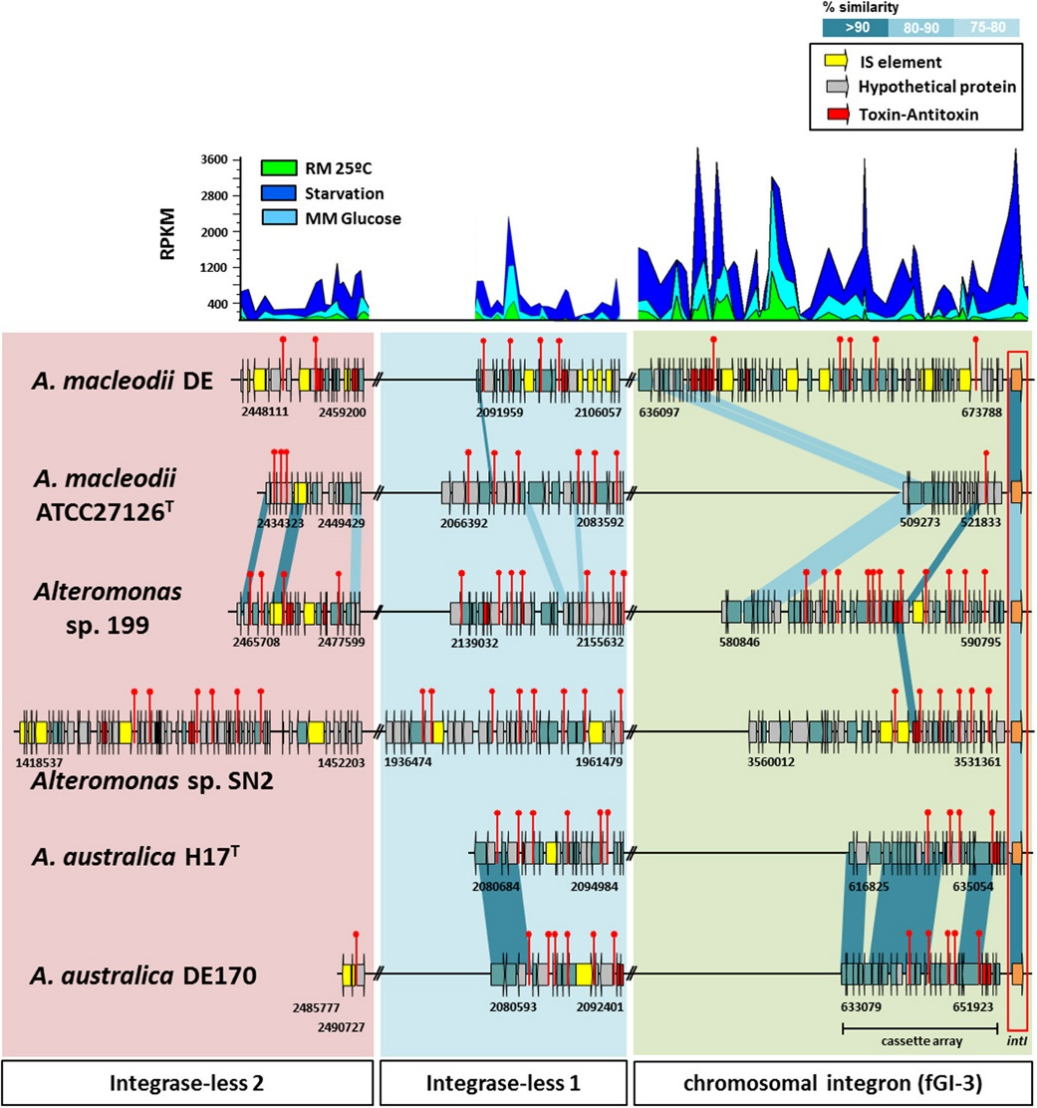
**Comparison of the integron gene cluster (fGI3).** Colored rectangles indicate the three different locations in the genome. Red asterisk indicated the position of the *attc* sequence. Gene expression data is expressed as RPKM (MM: Minimal Medium; RM: Rich Medium). The integron integrase is highlighted in orange and highlighted with a red rectangle.

fGI2 is an integron already described in *A. macleodii* [11] *.* Actually this region was proven to be among the most variable in this species. Specifically, the genomes of *A. macleodii* DE1 and UM7 which belong to the same deep ecotype clonal frame, differ in only 87 single nucleotide polymorphisms (SNPs) over the core genome and were identical even over the fGIs [11] had different gene cassettes in the integron. (Additional file 1: Figure S4). Integrons have widespread distribution among the genomes of marine bacteria (i.e. *Vibrionales*, *Alteromonadales* and *Pseudomonadales*) [29, 30]. In *A. australica* there is a different version, like in all the other genomes of *Alteromomas* described thus far. Integron identification is typically based on the presence of two parts. The first contains the elements responsible of the integration: an integrase gene (*intI*) that catalyzes the excision and integration of gene cassettes, a proximal primary recombination site (*attI*), multiple target-specific recombination sites (*attC*), and the promoter Pc that drives the transcription of the captured genes cassette [31]. The second part are the gene cassettes that are variable and responsible of the main differences among the strains. The integrase is the only gene conserved in all the fGI3 versions in *Alteromonas*. The presence of the five structural motifs of integron integrases (boxes I and II and patches I, II, and III) were well conserved (Additional file 1: Figure S5) identifying clearly this as a bona fide integron. We have built a phylogenetic tree (Additional file 1: Figure S6) with the *intI* genes from all the *Alteromonas* and the 104 sequences more similar to the *intI* gene of *A. australica* found in GenBank. The tree shows that, leaving aside most of the *Vibrio* species that form a homogeneous group, all the strains belonging to *A. macleodii* surface ecotype, *A. australica* and *Alteromonas* sp. SN2 were grouped together with the *Glaciecola* species *intI* while *A. macleodii* deep ecotype clustered with the corresponding gene of *Pseudoalteromonas* sp. As could be expected, the tree is markedly different from the phylogenomic tree of Figure 2, reflecting the horizontal transfer of these mobile elements. We have identified the *attC* site of the *Alteromonas* integron, a small region between 55 and 87 bp. This conserved sequence is located upstream of each gene cassette*.* The *attC* is better conserved than the *attI* [32] and has been well characterized in several species of *Vibrio* [33]. We have found also this *attC* sequence (>96% nucleotide identity) in the chromosome and plasmid of *Glaciecola* sp. 4H-3-7 + YE-5. Unexpectedly, we found *attC* sites in all the *Alteromonas* genomes at two other regions of the genome (Figure 3). However, the third region is not present in *A. australica* H17^T^ and strain DE170 only has two genes at this location. All these large arrays of cassettes found in the *Alteromonas* chromosome could be designated as a ‘super integron’ [34]. The insertion of new cassettes occurs more often next to the integrase than in the other two regions that do not have the integrase (Additional file 1: Figure S4). Considering the three regions, a total of 913 different genes were identified within all the *Alteromonas* integrons. Among these, almost 60% of them (572) were classified as hypothetical proteins making it impossible to infer any function. A similar proportion has been found in *Vibrio* species [30]. The second most abundant group of genes (12%) were assigned to toxin/antitoxin (TA) systems (ParDE, RelBE, HigAB and CcdAB) that have been suggested to be involved in the stabilizing of the integron gene cassettes [32] and 9% corresponded to IS elements normally found in the largest integrons. Most of TA systems in all *Alteromonas* strains are concentrated in the genome in two of the integron regions (Additional file 1: Figure S4). Although the promoter is normally embedded in the integrase sequence, we know from the data of the recent transcriptional analysis performed of the AltDE and AltDE1 genomes (Kimes et al., submitted; see Additional Methods), that all the three integron regions are expressed (Figure 3). Although the expression pattern is similar in all the conditions (Kimes et al., submitted), the expression level was higher under starvation than under nutrient-rich conditions (Figure 3), suggesting that the integron cassettes might help the bacteria to survive during stress conditions [35]. Although the members of the same phylogenomic clade appear to have more similar cassettes, in the region next to the integrase, the cassettes were totally different for each strain showing a highly dynamic nature. It is remarkable that the two *A. australica* isolates possess three nearly identical cassettes in the integrase region, in spite of their distant geographical origin and its high variability in all the other strains.

**Figure 4:**
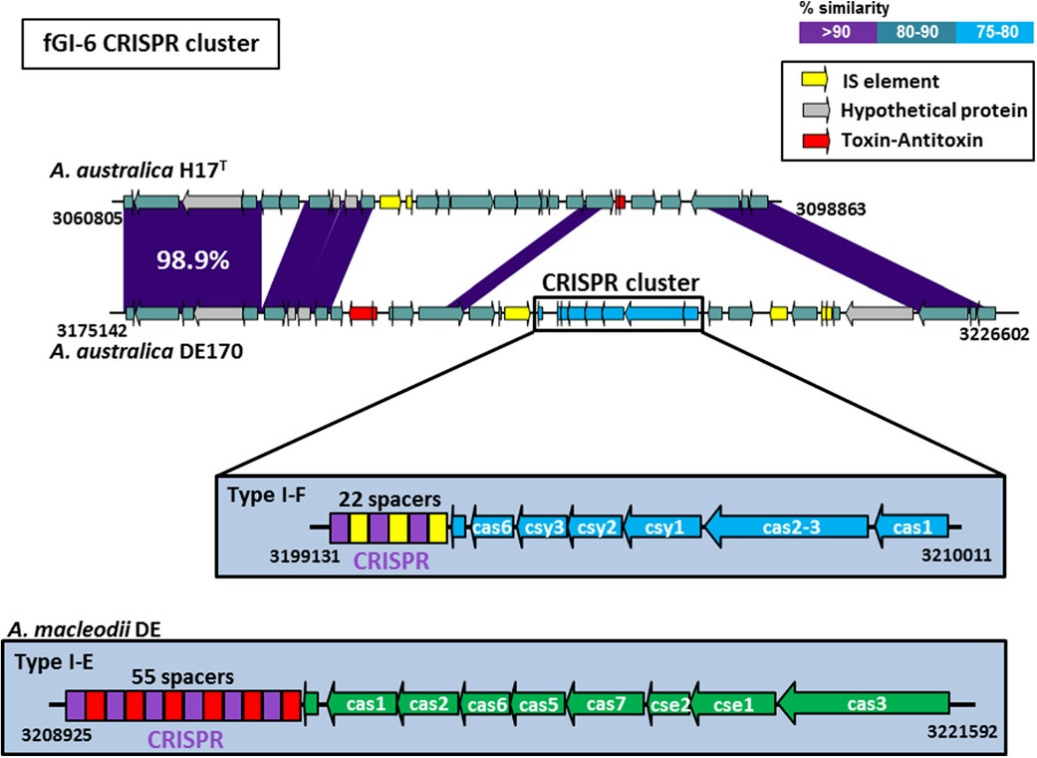
**Comparison of the gene content of the fGI6 in the** ***A. australica*** **genomes.** CRISPR-Cas system present in *A. autralica* DE170 and *A. macleodii* DE are shown in the highlighted blue rectangles.

Another fGI that is common to all *Alteromonas* species is fGI6 that, interestingly, in the case of *A. australica* DE170, contains the only CRISPR system described in *A. australica*. This system is used by bacteria and archaea as a defense against foreign DNA, either viral or plasmidic [36]. Twenty two spacers were identified by the CRISPR tool (CRISPRFinder; [37]) in the repeat-spacer array preceded by seven *cas* genes (*cas*6, *csy*3, *csy*2, *csy*1, *cas*2-3 and *cas*1). Based on the classification made by [38], DE170 CRISPR system belong to the subtype I-F, characterized by the fusion of *cas*2 and *cas*3 genes. Using the same method we could not find any CRISPR system in the H17^T^ genome. No match was obtained in the Blast searches of the 22 DE170 spacers against public databases. DE170 CRISPR cluster is flanked by IS elements, suggesting that it could be a mobile element as has been seen before in regions of *Escherichia coli* CRISPR gene clusters [39]. This is only the second CRISPR system found in *Alteromonas. A. macleodii* DE contains a CRISPR system of a different kind (subtype I-E) (Figure 4) [14] and none of the spacer sequences is shared between the two systems. The presence of different CRISPR subtypes in members of the same genus has also been described in *E. coli* strain collections [39]. Strain H17^T^ contains at the same location a totally different fGI and no trace of the CRISPR system. However, both *A. australica* isolates share a couple of genes coding for the TA system HipAB (Figure 4). It has been described that CRISPR systems and TA genes are often associated in genomic islands that have been named "defense islands" on the grounds of the anti-phage activities that both systems perform [40].

### Strain specific genomic islands

In addition, there are genomic islands that are found only in one of the strains. Five unique islands were found in strain H17^T^ and six in strain DE170 (Table 2). H17^T^ GI5 contains a gene cluster encoding a methanol/ethanol pyrroloquinoline quinone (PQQ)-dependent dehydrogenase and the putative *pqqABCDEF* operon that is used as a cofactor and have also shown to be an important antioxidant agent against oxidative damage [41]. This GI was located next to a single Leu-tRNA and at the end of this GI we found a direct repeat identical to the last 52 nucleotides of the tRNA 3′ end, evidence that the tRNA has acted as an insertion target of the GI in the same way as we found in fGI1. Methanol/ethanol dehydrogenase is a key enzyme used for growth in the presence of methanol/ethanol [42]. However, the growth on a one carbon substrate requires a complex metabolic machinery that has not been detected in *Alteromonas* yet. In fact, phenotypic analyses reveal that none of the two strains are able to grow in minimal medium with 1% of methanol (or ethanol) as sole carbon and energy source.

Some of the GIs appear to be associated to post-segregation killing systems. These systems have been mainly described as defense mechanisms against foreign DNA and involved in the stabilization of mobile elements [43]. GI2 in both strains encoded for a different classic type I restriction modification systems. Along the same lines, in the strain H17^T^ GI1 a complete gene cluster for a DNA backbone S-modification (phosphorothioation) was identified [44]. This system seems to act similarly to the classical restriction systems type I but protects DNA using phosphorothioation instead of methylation. The H17^T^ cluster is composed by seven genes, *dndBCDE* that are required for the DNA S-modification and another set (*dptFGH*) involved in the restriction of the unmodified DNA [45]. *A. australica* DE170 genome contains a mobilizable genomic island (MGI) (GI11). MGIs have been recently described as conjugative mobile elements that utilize the conjugation machinery of Integrative Conjugative Elements (ICE) or plasmids for their transfer. This GI could be considering other kind of "additive islands" [46]. In a recent study, five putative MGIs have been characterized in several strains of *A. macleodii* [11] sharing the same insertion point with the one of DE170.

Most of the heavy metal resistance genes in *Alteromonas* are clusterd in fGI1. In addition, strain DE170 have another GI containing a large cluster of genes that code for metal resistance related proteins. This 36 Kb region that was flanked by transposases was nearly identical to a region in the plasmid from *Glaciecola* sp. strain 4H-3-7 + YE-5 (pGLAAG01, 341 Kb). Interestingly, this plasmid was showed to be highly syntenic with the plasmid pAMDE1-300 from *A. macleodii* DE1 isolated from the same location as strain DE170 [9]. However, both plasmids have a large variable region with very different genes. Within the variable part in pGLAAG01 was this metal resistant GI found within the chromosome of strain DE170. In the case of pAMADE1-300 the variable region contains a hybrid NRPS-PKS cluster of 65 Kb [12] that was also found within the chromosome of two other *A. macleodii* strains [11]. These results illustrate the plasticity of these genomic elements that appear to cross easily the genus barrier.

GI4 and GI5 of DE170 appear to be involved in xylan degradation and cellulose biosynthesis. Xylan, a complex polysaccharide with a β-1,4-linked backbone of xylose, is the second most abundant plant cell wall polysaccharide [47]. The *xynTAB* and *xylREAB* clusters that code for the enzymes involved in the degradation and its conversion to D-xylulose 5-P, a pentose phosphate pathway intermediate, were all found in GI4 of DE170. Phenotypic analysis showed that strain D170 was indeed able to grow using xylan as a unique carbon and energy source while strain H17^T^ was not (see Methods). Also in DE170, the complete operon for bacterial cellulose synthesis (bcs) was found in GI10. This operon contains the genes that code for the two catalytic subunits, BcsA and BcsB, that are a transmembrane complex involved in the synthesis of cellulose from glucose and its and secretion. The other components (BscC and BscZ), are accessory proteins related to the regulation and the assembly of the complex [48]. Bacteria generally produce cellulose as a component of the extracellular matrix involved in biofilm formation and cell adhesion [49].

### Lysogenic phages

Both *A. australica* strains contain a genomic region with high similarity to CP4–57, a putative defective prophage described in *Escherichia coli* [50]. This prophage participates actively in *E. coli* biofilm development [51]. Like its *E. coli* counterpart, this putative prophage was found in both H17^T^ and DE170 genomes inserted at one tRNA (Leu) gene that contains a similar *att* site to the one described for prophage CP4-57 [51, 52]. A similar putative prophage was found at the equivalent location in three highly similar isolates of *A. macleodii* "deep ecotype" belonging to the same clonal frame and obtained from a deep sample (~3500 m) from the Urania Basin in the Ionian (Mediterranean) [11]. Actually, widely divergent versions of this prophage have been found in most *Alteromonas* genomes available, including the widely divergent SN2 (Additional file 1: Figure S7). In the *A. australica* strains the versions of this putative prophage were very divergent in sequence. The H17^T^ version was much more similar to the Urania isolates and the DE170 strain version was more similar to the isolate from the Yellow Sea sediment SN2 (Additional file 1: Figure S7).

**Figure 5:**
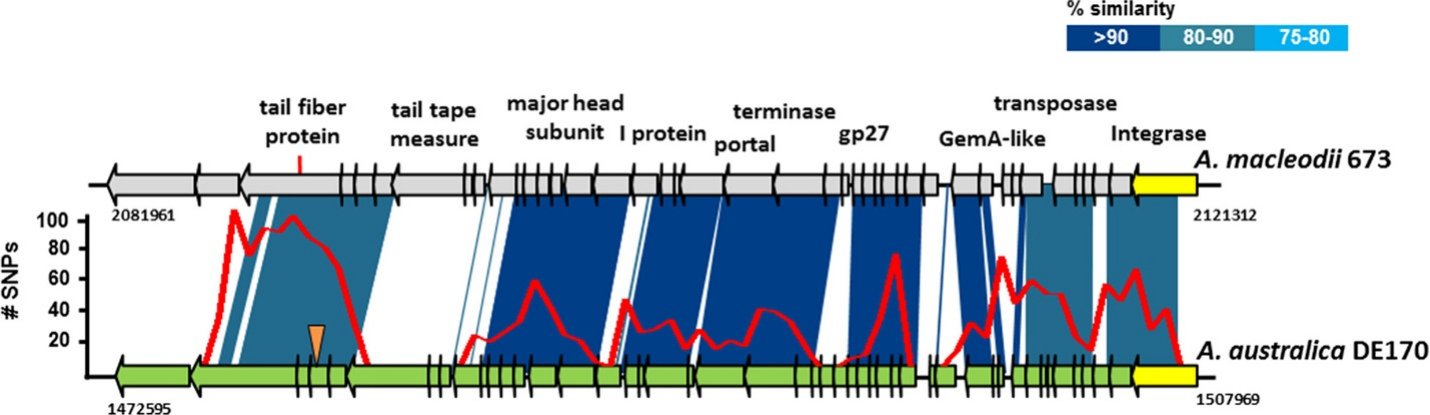
**Comparison of the Mu-like prophages found in** ***A. australica*** **DE170 and** ***A. macleodii*** **673.** Orange triangle indicates the protospacer of AltDE CRISPR system. The red line above DE170 indicates the number of SNPs in a 500-bp window.

In addition, in strain DE170, GI6 (47.5 Kb) (Figure 2 and Table 2) has all the hallmarks of a Mu-like lysogenic phage not present in H17^T^. DNA attachment sites *attL* and *attR* could be identified confirming this region as a prophage [53]. Interestingly, a highly related prophage (Figure 5) was previously described in the strain *A. macleodii* 673 (related only by a 74.1% ANI with DE170) [18]. This strain comes from the Western English Channel, showing the remarkably wide distribution of this phage. Furthermore, 28 of 30 consecutives nucleotides of the spacer 20 found in AltDE CRISPR system matched at 100% identity a hypothetical protein in the Mu-like prophage found in DE170 indicating that a highly related phage is also found in the Mediterranean. The protospacer region in these phages showed a large accumulation of SNPs in the comparison of both prophage genomes (Figure 5) indicating that it is highly variable. Actually, this region includes one ORF annotated as tail fiber protein, known to be involved in the determination of the host range specificity and often highly diversified [54]. Most of the variation in this gene was concentrated in the last (C-terminal) 200 nucleotides (Additional file 1: Figure S8) suggesting that it could be more important for the specificity. The other gene that showed a lower similarity coded for the tape measure protein that is the determinant of phage tail length, what might also indicate host specificity (in this case adapting the phage to different thickness of the host cell envelope). Both the presence of this spacer in the AltDE CRISPR and the variability found in these regions indicate that the prophage is widespread, often lytic and recognize different host strains.

### Recruitment from metagenomes

We have compared the recruitment of the genomes in different published marine metagenomes in order to compare the relative abundance of different species [18, 55]. These include both central North Pacific and North Atlantic gyres (Hawaii Ocean Time Series-HOTS and Bermuda Atlantic Time Series-BATS) metagenomes [56, 57] and the different collections of the Global Ocean Survey (GOS) [58] among others (see Methods). A restrictive cut-off of 98% of identity in 90% of the length of the environmental read was established to perform BLASTN comparisons against the genome of a representative of each species of *Alteromonas*. Unfortunately with the single exception of the deepest Mediterranean basin Matapan-Varilov [59] none of the *Alteromonas* genomes recruit significantly from marine metagenomic datasets (Additional file 1: Figure S9). From the Matapan-Varilov sample the *A. australica* genomes recruit similarly to *A. macleodii* DE, indicating a possible large particle habitat [14] also for this species. We also compared the genomes with the Dissolved Organic Matter (DOM), Deep Sea Water (DSW) and Dauphin Island Cubitainer Experiment (DICE) metatranscriptomes [6, 7, 60]. These three microcosms experiments were designed to analyze the transcriptional response of the bacterial community from ocean surface waters to adding dissolved organic matter (DOM and DICE) and deep seawater samples (DSW). Interestingly, all the species representative genomes (one for each since the method cannot distinguish differential recruitment within a single species, see Methods) recruit highly in these metatranscriptomes (Additional file 1: Figure S9).

## Discussion

The two genomes described here are the first from a species of *Alteromonas* formally described as different from *A. macleodii.* Actually the genome of strain SN2 also belongs to what certainly is a separate species, although it has not been formally described as such and remains as *Alteromonas* sp. We have also the added advantage that the two available genomes come from isolates obtained in very different locations (actually from nearly exact geographic antipodes) and habitats (surface coastal waters versus bathypelagic and off-shore). The first fact to underline is the remarkable similarity between the two genomes that actually recalls the diversity found for *A. macleodii* concurrent isolates. This supports the global distribution of *Alteromonas* species compounded with a high diversity of concurrent clones at every specific location. We posit that *Alteromonas* populations and actually most, if not all, prokaryotes live in complex consortia of multiple clones that have different gene complements, increasing the activities and environmental ranges in which the population can be successful [61, 62].

The core genome of all the strains of *Alteromonas* described to date (*ca*. 2000 genes) is relatively large (see below) although the ANI found among these microbes (Figure 2) indicates that they are widely divergent lineages as expected from strains from different species within the same genus. The core genome could shrink as new strains are sequenced. Particularly the species that live in cold environments such as *A. stellipolaris* isolated from Antarctic sea water [8], might have quite different gene pools reflecting their different habitats. As a reference, in the comparison of 7 species of the genus *Pseudomonas* which genome has a similar genome size to the genus *Alteromonas*, were found *ca*.1500 genes making up the core [63]. In the same way, 11 species of the genus *Glaciecola* that belong to the same family had 1257 genes [64]. Actually, from the ANI values *A. macleodii* could be in really composed of at least 3 species, the strains defined as the deep ecotype, the yellow sea isolate and the global surface ecotype with ANIs ranging from 73.7 to 80.6%. With this proviso we would be dealing with the equivalent of 5 different species genomes in this calculation of the *Alteromonas* core genome. The flexible part of the genome thus far would add up to *ca*. 13000 genes, also a typical value if is compared with the marine bacteria of the related genus *Glaciecola* (17276 gene families) [64] or the more than 17000 of the *Vibrio* pangenome [65].

A remarkable phenomenon that we have discovered is the continuum at the level of the genes found in the flexible genome across the genus. The fact that flexible genomic islands are found at equivalent genomic context, even when considering different species of the same genus, indicates that they are quite stable genomic features. In addition, we found remarkable examples of conservation in the flexible genome. For example, the presence of a nearly identical hydrogenase cluster shared by *A. macleodii* DE (Mediterranean Sea) and H17^T^ (Tasman Sea), or the presence of nearly identical integron cassettes in the two *A. australica* strains. This was expected from the mobile nature of these genetic elements. However, the absolute lack of geographic patterns was more unexpected [66]. Also, the conservation of these elements is nearly perfect, indicating that they have been exchanged recently, in spite of the large geographic distance. Some fGIs that are not mobile such as the flagellum glycosylation have also been found to be perfectly conserved in strains of distant geographic origins [11]. It seems that the core genome diverges more (at the level of SNPs) than the flexible one, probably because the latter is much more homogenized due to its frequent exchange, either by homologous or illegitimate recombination. In addition, flexible genes jump over the species barriers much more often than the core genome.

The recruitment, mostly from metatranscriptomes indicates that i) the genus *Alteromonas* is one of the most actively blooming microbes in sea water, ii) they have a worldwide distribution from the deep waters of the Mediterranean to the Pacific and Atlantic oceans and iii) they appear in complex consortia of multiple species. It was already known that *A. macleodii* lives in complex consortia of multiple clonal lineages that appear concurrently at any given sample [9]. A similar situation has been found for other marine microbes such as *Prochlorococcus* [62]. It would seem that the same applies to different species within the same genus. This adds to the plankton paradox [67] including even more diversity levels to the populations within the same communities.

## Conclusions

The two genomes of *A. australica* described here have a similar pattern of variation than the two isolates of *A. macleodii* isolated from the same location in the deep Mediterranean and previously described. Although the genomes are largely syntenic and with a high ANI over the homologous regions, large flexible genomic islands are markedly different in the two strains. This fGIs are of two types, i) the total replacement fGIs, in which the gene cluster in one strain is completely different in gene content from the other strain but with similar assigned function, and ii) the additive fGIs in which gene cassettes are added or deleted depending on the strains but retain some similar regions. It is remarkable that in spite of the large difference in ANI between *A. australica* and *A. macleodii* the fGIs are located at equivalent positions and with similar assigned functions (at least for the replacement type).

Like in the case of *A. macleodii*, metagenomic recruitments indicate that the different strains coexist at the same locations and have probably a global distribution in temperate and tropical latitudes. Both *A. macleodii* and *A. australica* are bloomers that increase their presence in fertilized mesocosms when an input of nutrients is provided.

## Methods

### Sample collection and sequencing

Details of isolation and origin of the *Alteromonas* strains using in this study have been described in Additional file 1: Figure S1. DNA was extracted by phenol-chloroform as described in [68] and checked for quality on a 1% agarose gel. The quantity was measured using Quant-iT® PicoGreen® dsDNA Reagent (Invitrogen). The genomes were sequenced using the IlluminaHiSeq 2000 (100-bp paired-end read) sequencing platform (Macrogen, Korea). The generated reads were trimmed and assembled *de novo* with VELVET, version 0.7.63 [69] using default parameters except for the *k*-mer that was 49. Combination of Geneious Pro 5.0.1 (with default parameters) using previously *Alteromonas* assembled genomes as a reference [9, 11, 18] and oligonucleotides designed from the sequence of the ends of assembled contigs were used in order to obtain one single closed contig.

### Genome annotation and analysis

The genomes were annotated using the NCBI PGAAP annotation pipeline ( http://www.ncbi.nlm.nih.gov/genome/annotation_prok/). The predicted protein sequences were compared using BLASTP to the NCBI nr protein database (e-value 10^-5^). ORFs smaller than 100 bp and without significant homology to other proteins were not considered. BioEdit was used to manipulate the sequences [70]. GC content was calculated using the EMBOSS tool *geecee* [71]. For comparative analyses, reciprocal BLASTN and TBLASTXs searches between the genomes were carried out, leading to the identification of regions of similarity, insertions and rearrangements. To allow the interactive visualization of genomic fragment comparisons Artemis v.12 [72], Artemis Comparison Tool ACTv.9 [73] were used to compare the genomes. The ANI between strains was calculated using JSpecies software package v1.2.1 using default parameters [74]. Sequences were aligned using MUSCLE version 3.6 [75] and ClustalW [76] and edited manually as necessary. Nucmer program in the MUMmer3+ package [77] was used to identify the indels and the SNPs between small regions of the genomes. Pan Genome Analysis Pipeline (PGAP) [78] was used to identify all of the orthologous pairs genes between *Alteromonas* genomes. The common dataset of shared genes among the strains was defined as their core genome. The total set of genes within the genomes was defined as the pan genome. The set of genes in each strain not shared with other strains was defined as unique genes.

### Phylogenetic analysis

To determine the exact phylogenetic relationship of the new isolates within the genus, phylogenetic analysis for all the *Alteromonas* members whose genomes were available were carried out (Additional file 1: Table S1). The tree was rooted using *Pseudoalteromonas atlantica* T6c (NC_008228.1) as an outgroup. The complete genomes were analyzed using TIGRfams to identify and concatenated all the conserved proteins. The concatenated proteins were aligned using Kalign [79] and a maximum likelihood tree was made using FastTree [80] using a JTT + CAT model and a gamma approximation.

### Recombination

The first step to analyze the recombination events among the *Alteromonas* strains was to make a multiple genome alignment. This was performed by using Mauve multiple alignment software (v2.3.1) [81]. ClonalFrame software v1.2 [20], a Bayesian inference method that reconstructs clonal relationships between the isolates in a sample, was used for estimating mutation and recombination rates. Three independent runs of ClonalFrame were performed each consisting of 100000 Markov chain Monte Carlo iterations. To assess the relative contribution of recombination and mutation, *r/m* and *ρ/θ* statistics were used.

### Recruitments of environmental collections

Genomes recruitments were carried out against some available marine metagenomes and metatranscriptomes [BATs (Bermuda Atlantic Time Series) [56], DICE (Dauphin Island Cubitainer Experiment) [60], DOM (Dissolved Organic Matter) [7], DSW (Deep Sea Water) [6], GOS (Global Ocean Survey) [58], HOTs (Hawaii Ocean Time Series) [57, 82], Marmara [83], MedDCM (Mediterranean Deep Chlorophyll Maximum) [84] and MVP (Matapan-Vavilov Deep) [59]]. BLASTN was carried out between the genome of a representative member of each *Alteromonas* species (*A. australica* DE170; *A. macleodii* ATCC27126^T^; *A. macleodii* DE; *Alteromonas* sp. ALT199 and *Alteromonas* sp. SN2) and the environmental databases. A restrictive cut-off of 98% of identity in 90% of the length of the environmental read was established to guarantee that only similarities at the species level were counted. The number of hits was normalized against the genomes and the database sizes.

### Phenotypic analyses

Growth assays in media with xylan as a sole a carbon and energy source were carried out in order to study if the presences of this gene cluster provided extra abilities in strain DE170. A 50 ml culture of both *A. australica* strains were grown in marine broth at 20°C until they reached to late exponential growth phase (Optical density 600 nm = 1.0). The cultures were washed twice using minimal medium without any carbon sources and then grown in minimal medium agar plates supplemented with 0.5, 1, 5 and 10 g l^-1^ of xylan from beechwood (Sigma; ×4252) at 20°C. The same washed cultures were used to analyze the Methanol/Ethanol dehydrogenase activity. To measure growth rate, cultures were grown in minimal medium with 1% ethanol or methanol as a sole carbon source in Erlenmeyer flasks with orbital shaking (200 rpm) and incubated at 20°C. Growth was measured by the optical density at 600 nm.

### Accession numbers

The genome sequences have been deposited in GenBank under the following bioproject accession numbers; PRJNA246143 for *A. australica* H17^T^ and PRJNA246140 for *A. australica* DE170.

## Electronic supplementary material

Additional file 1: Figure S1: *A. macleodii* isolates used in this study and their origin. **Figure S2.** Maximum-likelihood phylogenetic trees of randomly selected collinear blocks of sequence (size indicated under each tree). Members of different species were indicated with different color letters. In a black rectangle the core tree (as in Figure 2) is shown as a reference. *A. australica* DE170 genome was used to locate the position of the sequences used to perform the trees. **Figure S3.** Alignment of the fGI1 in some species of *Alteromonas.* Red arrows under the genomes indicate the 3’ end of the tRNA gene section that is duplicated. The plots above the genomes indicated the number of SNPs in a 500-bp window in comparison with the genome located on the top. The average and the total number of SNPs in the genome are indicated by a red dot line. **Figure S4.** Comparison of the DE1 and UM7 integron cluster. The integron integrase is marked with a red rectangle. Gene expression data (expressed as RPKM) is shown mapped to the *A. macleodii* DE1. **Figure S5.** Alignment of C-terminal integrases *Alteromonas* sequences. **Figure S6.** Phylogenetic tree of the *Alteromonas* integron integrases and some reference sequences similar to the *intI* gene of *A. australica* found in GenBank. Color code indicates members of the different species of *Alteromonas.* **Figure S7.** Putative Prophage CP4-57-like found in both *A. australica* and comparison to the similar prophages found in *other Alteromonas*. **Figure S8.** Alignment of the tail fiber protein from Mu-like prophages inserted in the *A. australica* DE170 and *A. macleodii* 673 genomes. **Figure S9.** *Alteromonas* species genomes relative recruitment of metagenomic reads at 98% identity and 90% coverage from some marine reference metagenomes. **Table S1.** Features of the reference genomes. (PDF 8 MB)

## References

[1] Sass AM, Sass H, Coolen MJL, Cypionka H, Overmann J (2001). Microbial communities in the chemocline of a hypersaline deep-sea Basin (Urania Basin, Mediterranean Sea). Appl Environ Microbiol.

[2] Baumann L, Baumann P, Mandel M, Allen RD (1972). Taxonomy of aerobic marine eubacteria. J Bacteriol.

[3] Acinas SG, Antón J, Rodríguez-Valera F (1999). Diversity of free-living and attached bacteria in offshore Western Mediterranean Waters as depicted by analysis of genes encoding 16S rRNA. Appl Environ Microbiol.

[4] García-Martínez J, Acinas SG, Massana R, Rodríguez-Valera F (2002). Prevalence and microdiversity of *Alteromonas macleodii*-like microorganisms in different oceanic regions. Environ Microbiol.

[5] López-López A, Bartual SG, Stal L, Onyshchenko O, Rodríguez-Valera F (2005). Genetic analysis of housekeeping genes reveals a deep-sea ecotype of *Alteromonas macleodii* in the Mediterranean Sea. Environ Microbiol.

[6] Shi Y, McCarren J, DeLong EF (2012). Transcriptional responses of surface water marine microbial assemblages to deep-sea water amendment. Environ Microbiol.

[7] McCarren J, Becker JW, Repeta DJ, Shi Y, Young CR, Malmstrom RR, Chisholm SW, DeLong EF (2010). Microbial community transcriptomes reveal microbes and metabolic pathways associated with dissolved organic matter turnover in the sea. Proc Natl Acad Sci.

[8] Van Trappen S, Tan T-L, Yang J, Mergaert J, Swings J (2004). Alteromonas stellipolaris sp. nov., a novel, budding, prosthecate bacterium from Antarctic seas, and emended description of the genus Alteromonas. Int J Syst Evol Microbiol.

[9] Gonzaga A, Martin-Cuadrado A-B, López-Pérez M, Megumi Mizuno C, García-Heredia I, Kimes NE, Lopez-García P, Moreira D, Ussery D, Zaballos M, Ghai R, Rodriguez-Valera F (2012). Polyclonality of concurrent natural populations of *Alteromonas macleodii*. Genome Biol Evol.

[10] Ivanova E, Ng H, Webb H, Kurilenko V, Zhukova N, Mikhailov V, Ponamoreva O, Crawford R (2013). Alteromonas australica sp. nov., isolated from the Tasman Sea. Antonie Van Leeuwenhoek.

[11] López-Pérez M, Gonzaga A, Rodriguez-Valera F (2013). Genomic diversity of "Deep Ecotype"*Alteromonas macleodii* isolates: evidence for Pan-Mediterranean Clonal Frames. Genome Biol Evol.

[12] Mizuno CM, Kimes NE, López-Pérez M, Ausó E, Rodriguez-Valera F, Ghai R (2013). A Hybrid NRPS-PKS gene cluster related to the Bleomycin family of Antitumor antibiotics in *Alteromonas macleodii* Strains. PLoS One.

[13] López-Pérez M, Martin-Cuadrado A-B, Rodriguez-Valera F (2014). Homologous recombination is involved in the diversity of replacement flexible genomic islands in aquatic prokaryotes. Front Genet.

[14] Ivars-Martinez E, Martin-Cuadrado A-B, D’Auria G, Mira A, Ferriera S, Johnson J, Friedman R, Rodriguez-Valera F (2008). Comparative genomics of two ecotypes of the marine planktonic copiotroph *Alteromonas macleodii* suggests alternative lifestyles associated with different kinds of particulate organic matter. ISME J.

[15] Math RK, Jin HM, Kim JM, Hahn Y, Park W, Madsen EL, Jeon CO (2012). Comparative genomics reveals adaptation by Alteromonas sp. SN2 to Marine Tidal-Flat conditions: cold tolerance and Aromatic Hydrocarbon metabolism. PLoS One.

[16] Gonzaga A, López-Pérez M, Martin-Cuadrado A-B, Ghai R, Rodriguez-Valera F (2012). Complete genome sequence of the Copiotrophic Marine Bacterium *Alteromonas macleodii* Strain ATCC 27126^T^. J Bacteriol.

[17] Gauthier G, Gauthier M, Christen R (1995). Phylogenetic analysis of the Genera Alteromonas, Shewanella, and Moritella using genes coding for small-subunit rRNA Sequences and Division of the Genus Alteromonas into Two Genera, Alteromonas (Emended) and Pseudoalteromonas gen. nov., and proposal of twelve new species combinations. Int J Syst Bacteriol.

[18] López-Pérez M, Gonzaga A, Martin-Cuadrado A-B, Onyshchenko O, Ghavidel A, Ghai R, Rodriguez-Valera F (2012). Genomes of surface isolates of *Alteromonas macleodii*: the life of a widespread marine opportunistic copiotroph. Sci Rep.

[19] Didelot X, Falush D (2007). Inference of bacterial microevolution using Multilocus sequence data. Genetics.

[20] Didelot X, Lawson D, Darling A, Falush D (2010). Inference of homologous recombination in bacteria using whole-genome sequences. Genetics.

[21] Read TD, Joseph SJ, Didelot X, Liang B, Patel L, Dean D (2013). Comparative analysis of *Chlamydia psittaci* genomes reveals the recent emergence of a pathogenic lineage with a broad host range. mBio.

[22] Coleman ML, Sullivan MB, Martiny AC, Steglich C, Barry K, DeLong EF, Chisholm SW (2006). Genomic islands and the ecology and evolution of *Prochlorococcus*. Science.

[23] Dufresne A, Ostrowski M, Scanlan D, Garczarek L, Mazard S, Palenik B, Paulsen I, de Marsac N, Wincker P, Dossat C, Ferriera S, Johnson J, Post AF, Hess WR, Partensky F (2008). Unraveling the genomic mosaic of a ubiquitous genus of marine cyanobacteria. Genome Biol.

[24] Lopez-Perez M, Ghai R, Leon M, Rodriguez-Olmos A, Copa-Patino J, Soliveri J, Sanchez-Porro C, Ventosa A, Rodriguez-Valera F (2013). Genomes of "Spiribacter", a streamlined, successful halophilic bacterium. BMC Genomics.

[25] Pasic L, Rodriguez-Mueller B, Martin-Cuadrado A-B, Mira A, Rohwer F, Rodriguez-Valera F (2009). Metagenomic islands of hyperhalophiles: the case of *Salinibacter ruber*. BMC Genomics.

[26] Weyman PD, Smith HO, Xu Q (2011). Genetic analysis of the *Alteromonas macleodii* [NiFe]-hydrogenase. FEMS Microbiol Lett.

[27] Weyman PD, Vargas WA, Tong Y, Yu J, Maness P-C, Smith HO, Xu Q (2011). Heterologous expression of *Alteromonas macleodii* and *Thiocapsa roseopersicina* [NiFe] Hydrogenases in *Synechococcus elongatus*. PLoS One.

[28] Klippel B, Lochner A, Bruce DC, Davenport KW, Detter C, Goodwin LA, Han J, Han S, Land ML, Mikhailova N, Nolan M, Pennacchio L, Pitluck S, Tapia R, Woyke T, Wiebusch S, Basner A, Abe F, Horikoshi K, Keller M, Antranikian G (2011). Complete genome sequence of the marine, cellulose and xylan degrading bacterium *Glaciecola* sp. 4H-3-7 + YE-5. J Bacteriol.

[29] Mazel D (2006). Integrons: agents of bacterial evolution. Nat Rev Micro.

[30] Boucher Y, Labbate M, Koenig JE, Stokes HW (2007). Integrons: mobilizable platforms that promote genetic diversity in bacteria. Trends Microbiol.

[31] Hall RM, Collis CM (1995). Mobile gene cassettes and integrons: capture and spread of genes by site-specific recombination. Mol Microbiol.

[32] Cambray G, Guerout A-M, Mazel D (2010). Integrons. Annu Rev Genet.

[33] Biskri L, Bouvier M, Guérout A-M, Boisnard S, Mazel D (2005). Comparative study of class 1 Integron and *Vibrio cholerae* Superintegron Integrase activities. J Bacteriol.

[34] Rowe-Magnus DA, Guérout A-M, Mazel D (1999). Super-integrons. Res Microbiol.

[35] Guerin É, Cambray G, Sanchez-Alberola N, Campoy S, Erill I, Da Re S, Gonzalez-Zorn B, Barbé J, Ploy M-C, Mazel D (2009). The SOS response controls integron recombination. Science.

[36] Bhaya D, Davison M, Barrangou R (2011). CRISPR-cas systems in bacteria and archaea: versatile small RNAs for adaptive defense and regulation. Annu Rev Genet.

[37] Grissa I, Vergnaud G, Pourcel C (2007). CRISPRFinder: a web tool to identify clustered regularly interspaced short palindromic repeats. Nucleic Acids Res.

[38] Makarova KS, Haft DH, Barrangou R, Brouns SJJ, Charpentier E, Horvath P, Moineau S, Mojica FJM, Wolf YI, Yakunin AF, van der Oost J, Koonin EV (2011). Evolution and classification of the CRISPR–Cas systems. Nat Rev Micro.

[39] Almendros C, Mojica FJM, Díez-Villaseñor C, Guzmán NM, García-Martínez J (2014). CRISPR-Cas functional module exchange in *Escherichia coli*. mBio.

[40] Koonin EV, Makarova KS (2009). CRISPR-Cas: an adaptive immunity system in prokaryotes. F1000 Biol Rep.

[41] Misra HS, Rajpurohit YS, Khairnar NP (2012). Pyrroloquinoline-quinone and its versatile roles in biological processes. J Biosci.

[42] Anthony C (2004). The quinoprotein dehydrogenases for methanol and glucose. Arch Biochem Biophys.

[43] Vasu K, Nagaraja V (2013). Diverse functions of restriction-modification systems in addition to cellular defense. Microbiol Mol Biol Rev.

[44] Zhou X, He X, Liang J, Li A, Xu T, Kieser T, Helmann JD, Deng Z (2005). A novel DNA modification by sulphur. Mol Microbiol.

[45] Xu T, Yao F, Zhou X, Deng Z, You D (2010). A novel host-specific restriction system associated with DNA backbone S-modification in *Salmonella*. Nucleic Acids Res.

[46] Daccord A, Ceccarelli D, Rodrigue S, Burrus V (2013). Comparative analysis of mobilizable genomic islands. J Bacteriol.

[47] Prade RA (1996). Xylanases: from biology to biotechnology. Biotechnol Genet Eng Rev.

[48] Römling U (2002). Molecular biology of cellulose production in bacteria. Res Microbiol.

[49] Ude S, Arnold DL, Moon CD, Timms-Wilson T, Spiers AJ (2006). Biofilm formation and cellulose expression among diverse environmental *Pseudomonas* isolates. Environ Microbiol.

[50] Kirby JE, Trempy JE, Gottesman S (1994). Excision of a P4-like cryptic prophage leads to Alp protease expression in *Escherichia coli*. J Bacteriol.

[51] Wang X, Kim Y, Wood TK (2009). Control and benefits of CP4-57 prophage excision in *Escherichia coli* biofilms. ISME J.

[52] Hauser MA, Scocca JJ (1992). Site-specific integration of the *Haemophilus influenzae* bacteriophage HP1: location of the boundaries of the phage attachment site. J Bacteriol.

[53] Zhou Y, Liang Y, Lynch KH, Dennis JJ, Wishart DS (2011). PHAST: a fast phage search tool. Nucleic Acids Res.

[54] Rodriguez-Valera F, Mizuno CM, Ghai R (2014). Tales from a thousand and one phages. Bacteriophage.

[55] Xia LC, Cram JA, Chen T, Fuhrman JA, Sun F (2011). Accurate genome relative abundance estimation based on shotgun metagenomic reads. PLoS One.

[56] Coleman ML, Chisholm SW (2010). Ecosystem-specific selection pressures revealed through comparative population genomics. Proc Natl Acad Sci.

[57] DeLong EF, Preston CM, Mincer T, Rich V, Hallam SJ, Frigaard N-U, Martinez A, Sullivan MB, Edwards R, Brito BR, Chisholm SW, Karl DM (2006). Community genomics among stratified microbial assemblages in the ocean’s interior. Science.

[58] Rusch DB, Halpern AL, Sutton G, Heidelberg KB, Williamson S, Yooseph S, Wu D, Eisen JA, Hoffman JM, Remington K, Beeson K, Tran B, Smith H, Baden-Tillson H, Stewart C, Thorpe J, Freeman J, Andrews-Pfannkoch C, Venter JE, Li K, Kravitz S, Heidelberg JF, Utterback T, Rogers YH, Falcón LI, Souza V, Bonilla-Rosso G, Eguiarte LE, Karl DM, Sathyendranath S (2007). The *sorcerer II* global ocean sampling expedition: northwest Atlantic through eastern tropical pacific. PLoS Biol.

[59] Smedile F, Messina E, La Cono V, Tsoy O, Monticelli LS, Borghini M, Giuliano L, Golyshin PN, Mushegian A, Yakimov MM (2013). Metagenomic analysis of hadopelagic microbial assemblages thriving at the deepest part of Mediterranean Sea, matapan-vavilov deep. Environ Microbiol.

[60] Howard EC, Sun S, Reisch CR, del Valle DA, Bürgmann H, Kiene RP, Moran MA (2011). Changes in dimethylsulfoniopropionate demethylase gene assemblages in response to an induced phytoplankton bloom. Appl Environ Microbiol.

[61] Rodriguez-Valera F, Martin-Cuadrado A-B, Rodriguez-Brito B, Pasic L, Thingstad TF, Rohwer F, Mira A (2009). Explaining microbial population genomics through phage predation. Nat Rev Micro.

[62] Kashtan N, Roggensack SE, Rodrigue S, Thompson JW, Biller SJ, Coe A, Ding H, Marttinen P, Malmstrom RR, Stocker R, Follows MJ, Stepanauskas R, Chisholm SW (2014). Single-cell genomics reveals hundreds of coexisting subpopulations in wild *Prochlorococcus*. Science.

[63] Loper JE, Hassan KA, Mavrodi DV, Davis EW, Lim CK, Shaffer BT, Elbourne LDH, Stockwell VO, Hartney SL, Breakwell K, Henkels MD, Tetu SG, Rangel LI, Kidarsa TA, Wilson NL, van de Mortel JE, Song C, Blumhagen R, Radune D, Hostetler JB, Brinkac LM, Durkin AS, Kluepfel DA, Wechter WP, Anderson AJ, Kim YC, Pierson LS, Pierson EA, Lindow SE, Kobayashi DY (2012). Comparative genomics of plant-associated *Pseudomonas* spp.: insights into diversity and inheritance of traits involved in Multitrophic interactions. PLoS Genet.

[64] Qin QL, Xie BB, Yu Y, Shu YL, Rong JC, Zhang YJ, Zhao DL, Chen XL, Zhang XY, Chen B, Zhou BC, Zhang YZ (2013). Comparative genomics of the marine bacterial genus *Glaciecola* reveals the high degree of genomic diversity and genomic characteristic for cold adaptation. Environ Microbiol.

[65] Lukjancenko O, Ussery DW (2014). Vibrio chromosome-specific families. Front Microbiol.

[66] Elsaied H, Stokes HW, Kitamura K, Kurusu Y, Kamagata Y, Maruyama A (2011). Marine integrons containing novel integrase genes, attachment sites, *attI*, and associated gene cassettes in polluted sediments from Suez and Tokyo bays. ISME J.

[67] Hutchinson GE (1961). The paradox of the plankton. Am Nat.

[68] Neumann B, Pospiech A, Schairer HU (1992). Rapid isolation of genomic DNA from gram-negative bacteria. Trends Genet.

[69] Zerbino DR, Birney E (2008). Velvet: algorithms for de novo short read assembly using de bruijn graphs. Genome Res.

[70] Hall TA (1999). BioEdit: a user-friendly biological sequence alignment editor and analysis program for Windows 95/98/NT. Nucleic Acids Symp Ser.

[71] Rice P, Longden I, Bleasby A (2000). EMBOSS: the European Molecular Biology Open Software Suite. Trends Genet.

[72] Rutherford K, Parkhill J, Crook J, Horsnell T, Rice P, Rajandream M-A, Barrell B (2000). Artemis: sequence visualization and annotation. Bioinformatics.

[73] Carver TJ, Rutherford KM, Berriman M, Rajandream M-A, Barrell BG, Parkhill J (2005). ACT: the Artemis comparison tool. Bioinformatics.

[74] Richter M, Rosselló-Móra R (2009). Shifting the genomic gold standard for the prokaryotic species definition. Proc Natl Acad Sci.

[75] Edgar R (2004). MUSCLE: a multiple sequence alignment method with reduced time and space complexity. BMC Bioinformatics.

[76] Thompson JD, Higgins DG, Gibson TJ (1994). CLUSTAL W: improving the sensitivity of progressive multiple sequence alignment through sequence weighting, position-specific gap penalties and weight matrix choice. Nucleic Acids Res.

[77] Kurtz S, Phillippy A, Delcher A, Smoot M, Shumway M, Antonescu C, Salzberg S (2004). Versatile and open software for comparing large genomes. Genome Biol.

[78] Zhao Y, Wu J, Yang J, Sun S, Xiao J, Yu J (2012). PGAP: pan-genomes analysis pipeline. Bioinformatics.

[79] Lassmann T, Sonnhammer E (2005). Kalign - an accurate and fast multiple sequence alignment algorithm. BMC Bioinformatics.

[80] Price MN, Dehal PS, Arkin AP (2010). FastTree 2 – approximately maximum-likelihood trees for large alignments. PLoS One.

[81] Darling ACE, Mau B, Blattner FR, Perna NT (2004). Mauve: multiple alignment of conserved genomic sequence with rearrangements. Genome Res.

[82] Konstantinidis KT, Braff J, Karl DM, DeLong EF (2009). Comparative metagenomic analysis of a microbial community residing at a depth of 4,000 meters at station ALOHA in the north pacific subtropical gyre. Appl Environ Microbiol.

[83] Quaiser A, Zivanovic Y, Moreira D, Lopez-Garcia P (2011). Comparative metagenomics of bathypelagic plankton and bottom sediment from the Sea of Marmara. ISME J.

[84] Ghai R, Martin-Cuadrado A-B, Molto AG, Heredia IG, Cabrera R, Martin J, Verdu M, Deschamps P, Moreira D, Lopez-Garcia P, Mira A, Rodriguez-Valera F (2010). Metagenome of the Mediterranean deep chlorophyll maximum studied by direct and fosmid library 454 pyrosequencing. ISME J.

