# Supplementary material for: Genomes of Alteromonas australica, a world apart
Source: BMC Genomics. 2014 Jun 18;15(1):483. doi: 10.1186/1471-2164-15-483 (PMC4119200; doi:10.1186/1471-2164-15-483)
Supplement: Supplementary file 1 — Additional file 1: Figure S1: A. macleodii isolates used in this study and their origin. Figure S2. Maximum-likelihood phylogenetic trees of randomly selected collinear blocks of sequence (size indicated under each tree). Members of different species were indicated with different color letters. In a black rectangle the core tree (as in Figure 2) is shown as a reference. A. australica DE170 genome was used to locate the position of the sequences used to perform the trees. Figure S3. Alignment of the fGI1 in some species of Alteromonas. Red arrows under the genomes indicate the 3’ end of the tRNA gene section that is duplicated. The plots above the genomes indicated the number of SNPs in a 500-bp window in comparison with the genome located on the top. The average and the total number of SNPs in the genome are indicated by a red dot line. Figure S4. Comparison of the DE1 and UM7 integron cluster. The integron integrase is marked with a red rectangle. Gene expression data (expressed as RPKM) is shown mapped to the A. macleodii DE1. Figure S5. Alignment of C-terminal integrases Alteromonas sequences. Figure S6. Phylogenetic tree of the Alteromonas integron integrases and some reference sequences similar to the intI gene of A. australica found in GenBank. Color code indicates members of the different species of Alteromonas. Figure S7. Putative Prophage CP4-57-like found in both A. australica and comparison to the similar prophages found in other Alteromonas. Figure S8. Alignment of the tail fiber protein from Mu-like prophages inserted in the A. australica DE170 and A. macleodii 673 genomes. Figure S9. Alteromonas species genomes relative recruitment of metagenomic reads at 98% identity and 90% coverage from some marine reference metagenomes. Table S1. Features of the reference genomes. (PDF 8 MB) [file 12864_2014_6297_MOESM1_ESM.pdf]

Genomes of *Alteromonas australica*, a world apart

Mario López-Pérez[1]

Email: mario.lopezp@umh.es

Aitor Gonzaga[1]

Email: agonzaga.molto@gmail.com

Elena P. Ivanova[2]

Email: eivanova@swin.edu.au

and Francisco Rodriguez-Valera[1*]

Email: frvalera@umh.es

[1]Evolutionary Genomics Group, División de Microbiología, Universidad Miguel Hernández, Apartado 18, San Juan 03550, Alicante, Spain

[2] Swinburne University of Technology, PO Box 218, Hawthorn, VIC 3122, Australia

*Address correspondence to Francisco Rodriguez-Valera, frvalera@umh.es

Evolutionary Genomics Group, División de Microbiología, Universidad Miguel Hernández, Apartado 18, San Juan 03550, Alicante, Spain

Phone +34-965919313, Fax +34-965 919457

Running Head: Genomes of *Alteromonas australica*

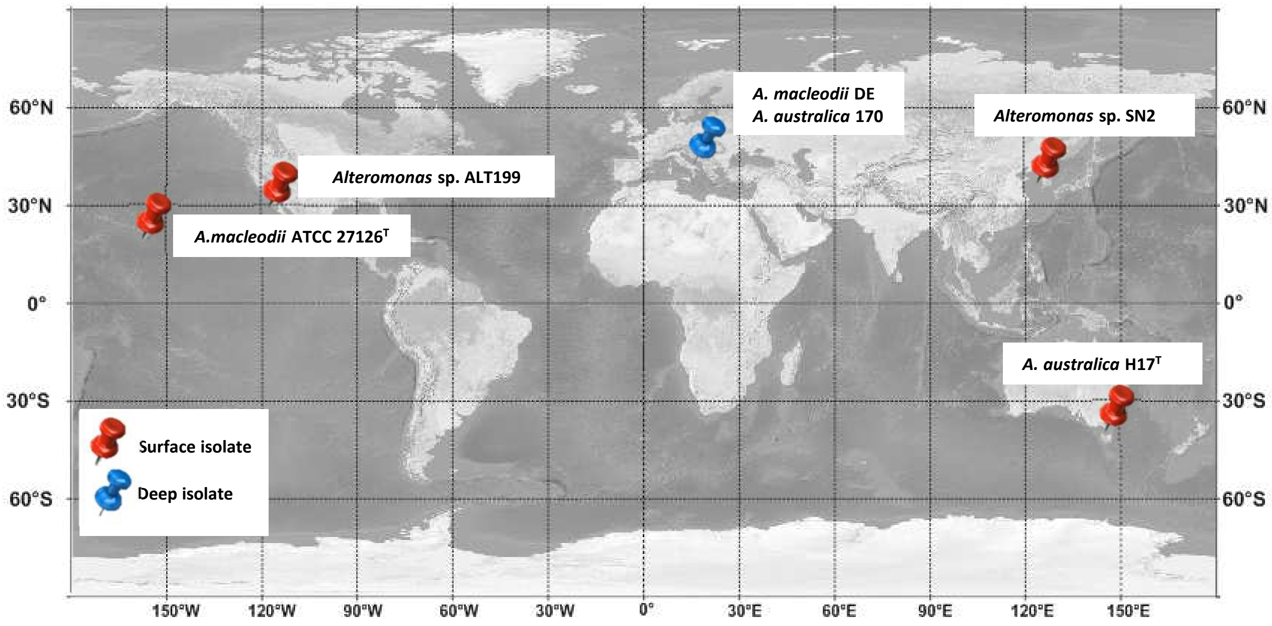

Alteromonas strain	Origen isolated	Location	Date	Depth of Sample (m)	Reference
***A. macleodii* DE**	Adriatic Sea	41°37′N, 17°22′E	2003	1000	López-López et al, 2005
***A. australica* DE170**	Adriatic Sea	41°37′N, 17°22′E	2003	1000	López-López et al, 2005
***A. macleodii* ATCC27126^{T}**	Pacific Ocean	21º17´23´´N 157º58´34´´W	1972	Surface	Baumann et al, 1972
***Alteromonas* sp. SN2**	Yellow Sea	36°48′50´´N, 126°11′9´´E	2007	Surface	Math et al, 2012
***A .australica* H17^{T}**	Tasman Sea	37º52´10´´S 144º58´27´´E	2007	Surface	Ivanova et al, 2013
***Alteromonas* sp. ALT199**	Pacific Ocean	32º44´50´´N 117º15´19´´W	--	Surface	--

Additional Figure S1.- *A. macleodii* isolates used in this study and their origin

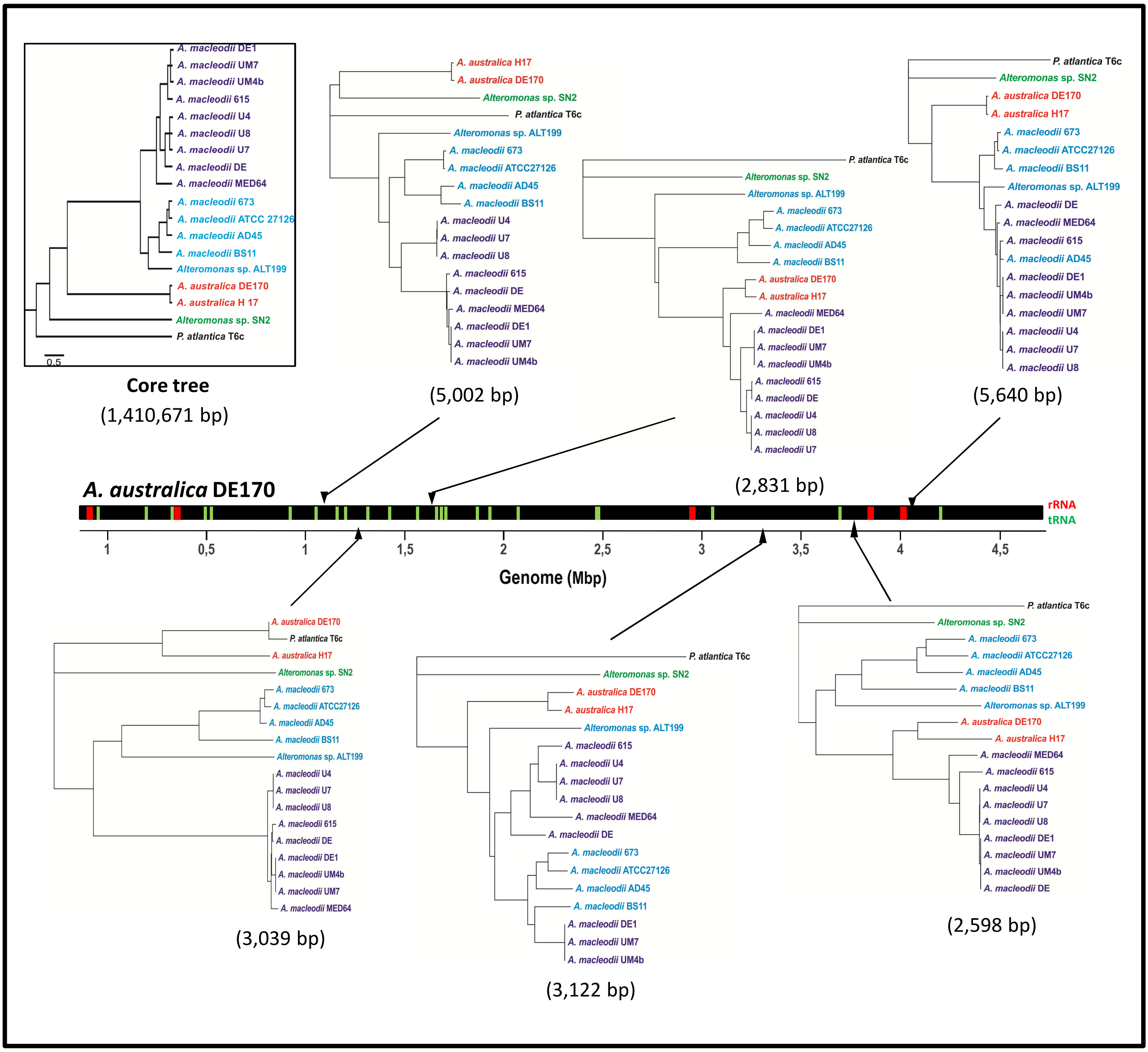

Additional Figure S2.- Maximum-likelihood phylogenetic trees of randomly selected collinear blocks of sequence (size indicated under each tree). *Pseudoalteromonas atlantica* T6c was used as an outgroup. Members of different species were indicated with different color letters. To the left in a black rectangle the core tree (as in Figure 1) is shown as a reference. *A. australica* DE170 genome was used to locate the position of the sequences used to perform the trees

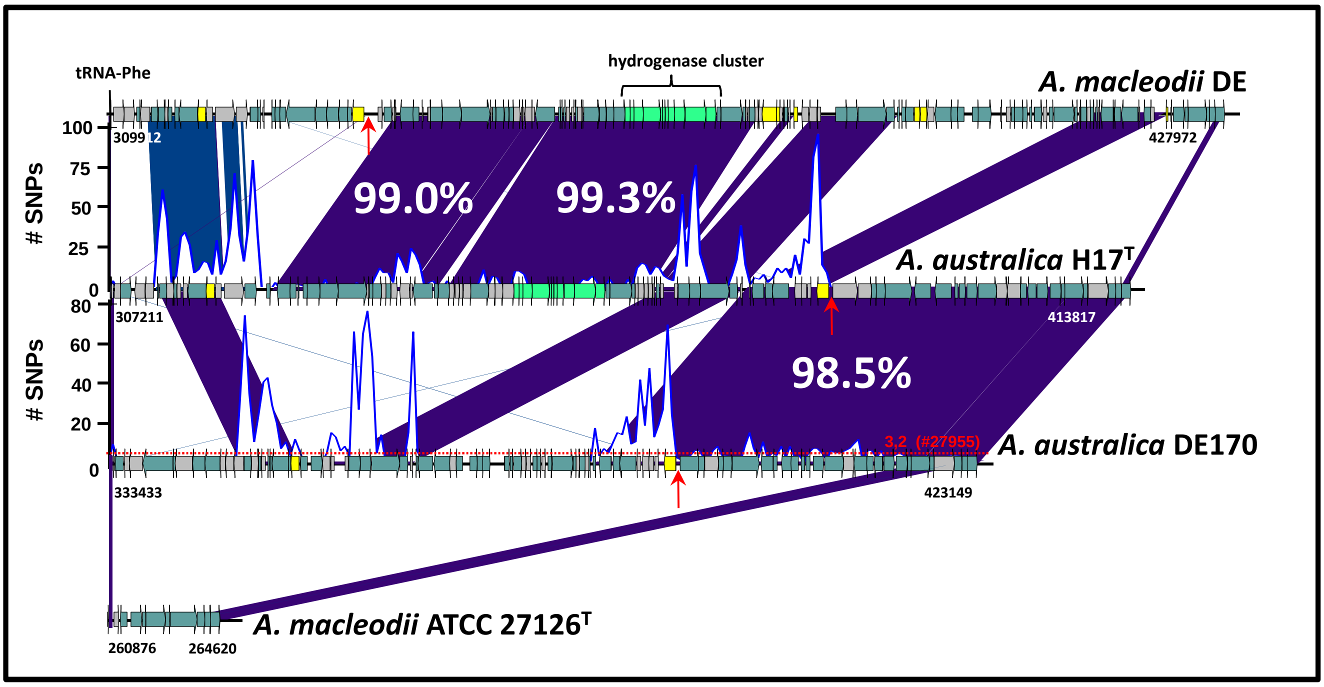

Additional Figure S3.- Alignment of the metal–resistance-related region in some species of *Alteromonas*. Red arrows under the genomes indicate the 3' end of the tRNA gene section that is duplicated and represents a hall mark of an integration event. The plots above the genomes indicated the number of SNPs in a 500-bp window in comparison with the genome located on the top. The average and the total number of SNPs in the genome are indicated by a red dot line.

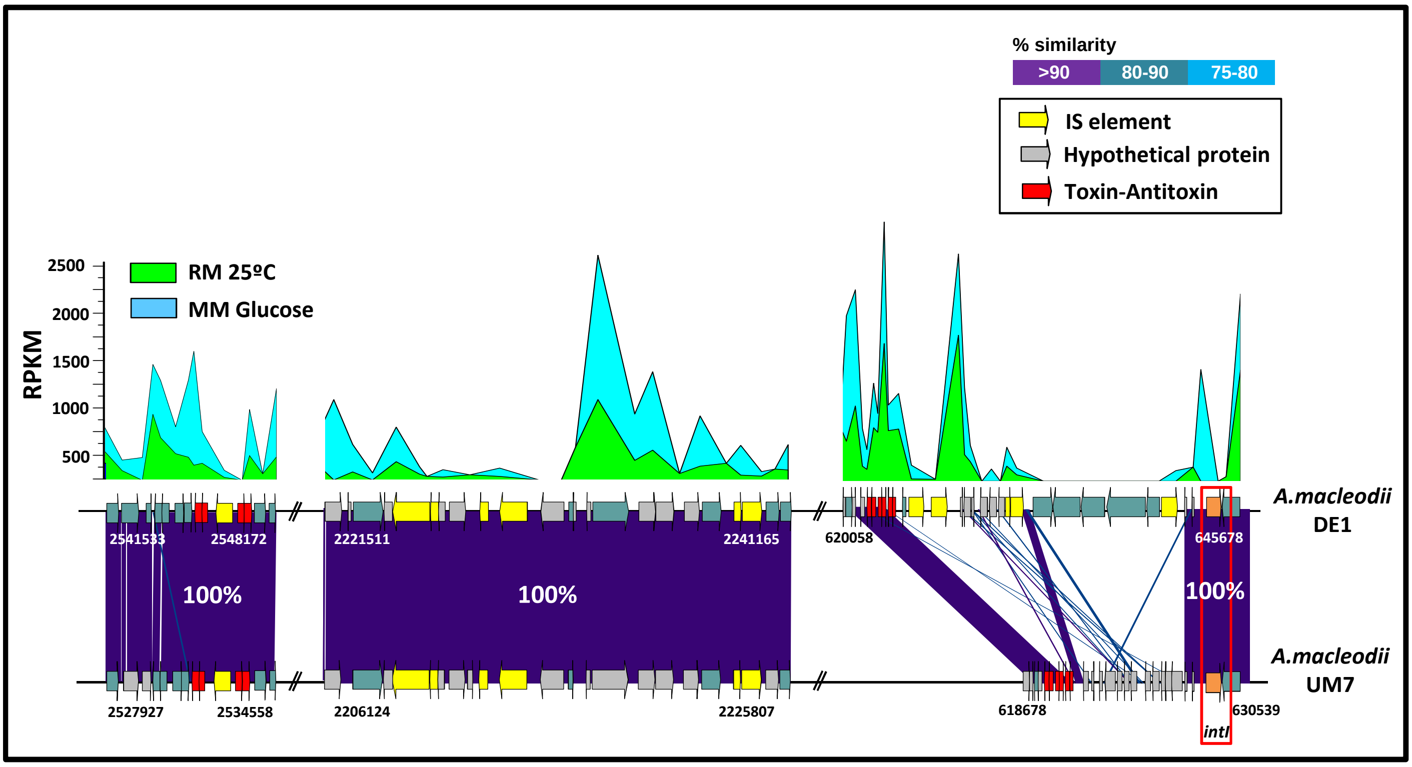

Additional Figure S4.- Comparison of the closest strains of *A. macleodii* sequenced yet by sequence similarity DE1 and UM7 integron cluster. The integron integrase is highlighted in orange and marked with a red rectangle. Gene expression data (expressed as RPKM) is shown mapped to the *A. macleodii* DE1.

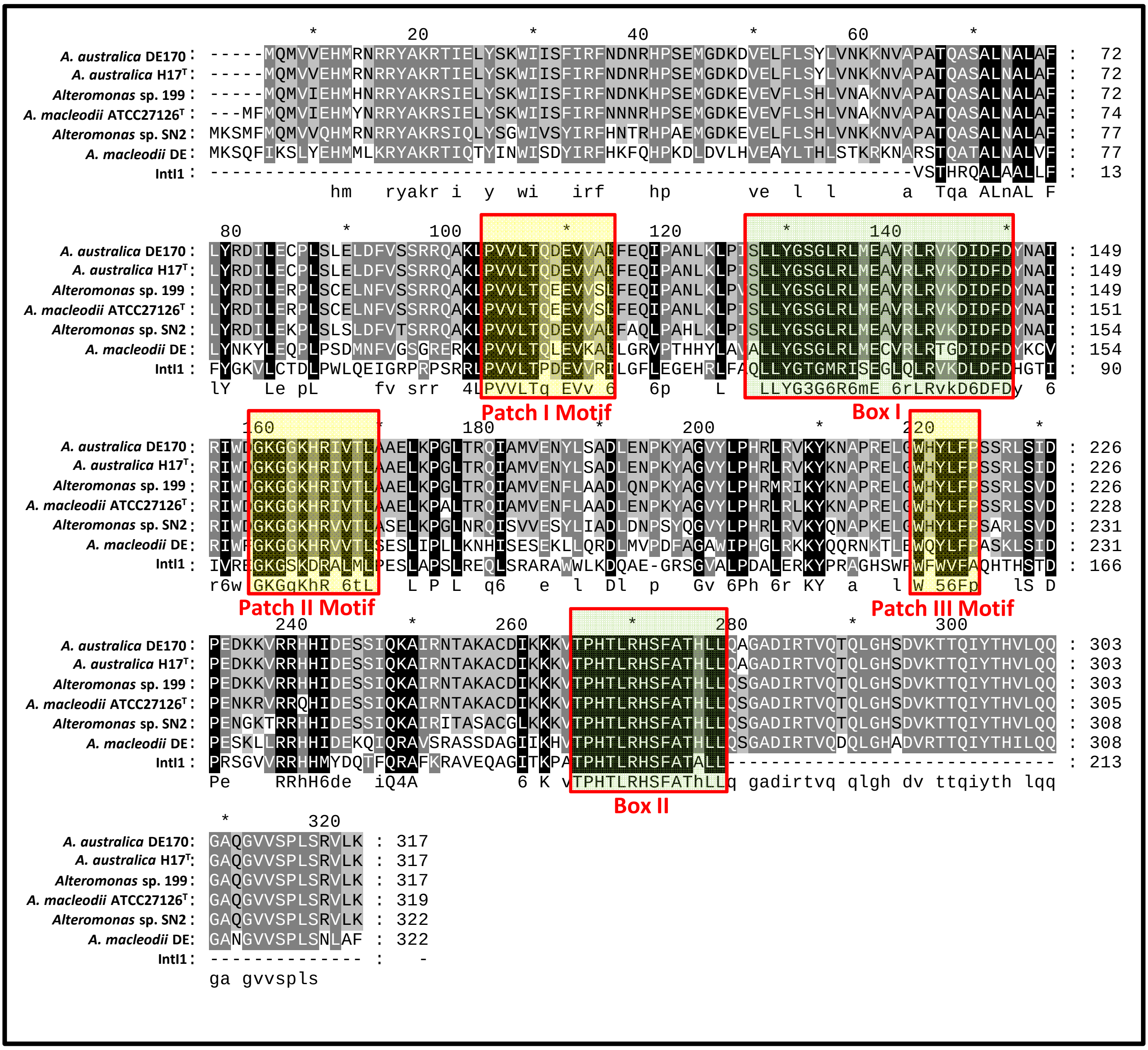

Additional Figure S5.- Alignment of C-terminal integrases *Alteromonas* sequences

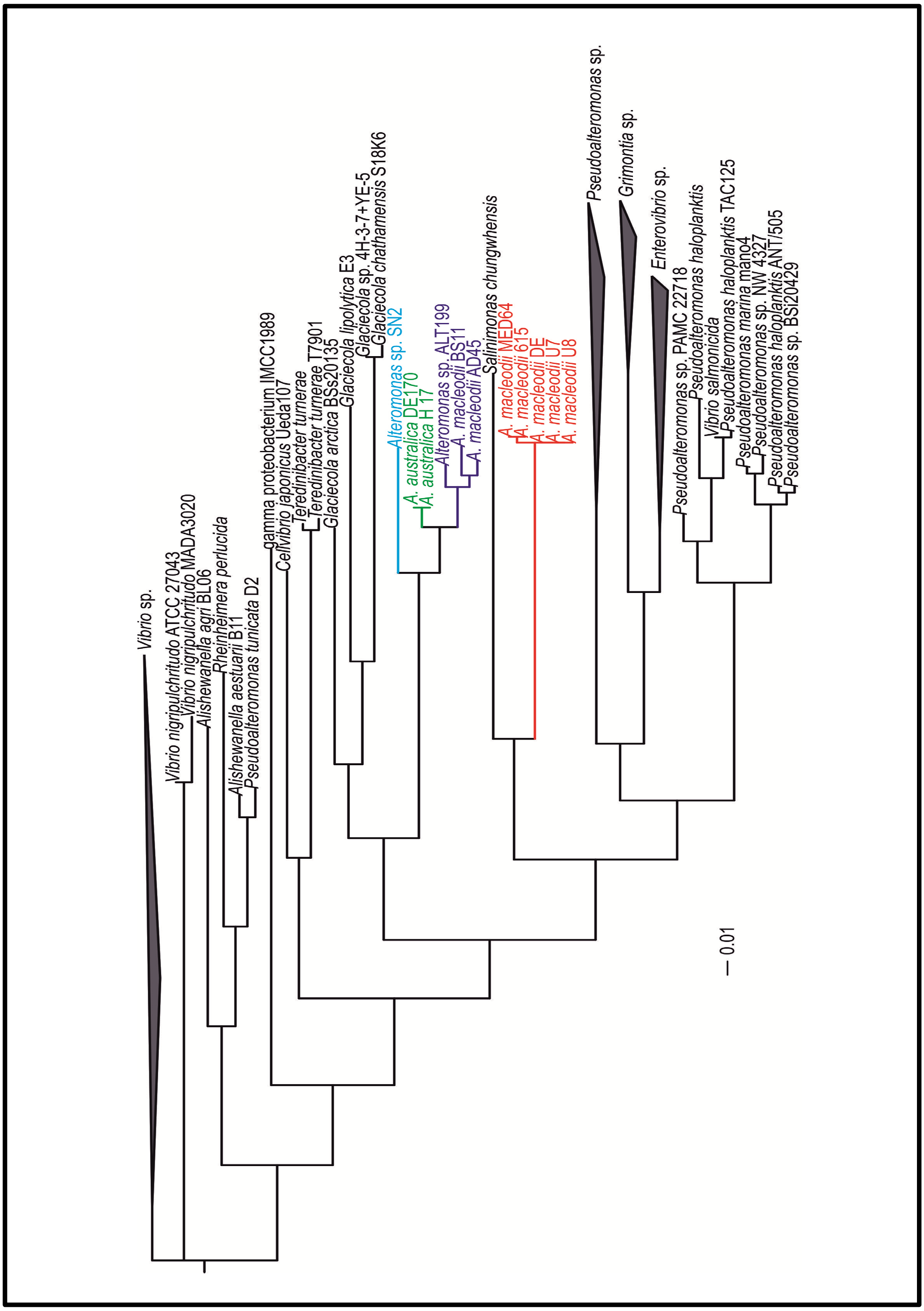

Additional Figure S6.- Phylogenetic tree of the *Alteromonas* integron integrases and some reference sequences similar to the *intI* gene of *A. australica* found in GenBank. Color code indicates members of the different species of *Alteromonas*

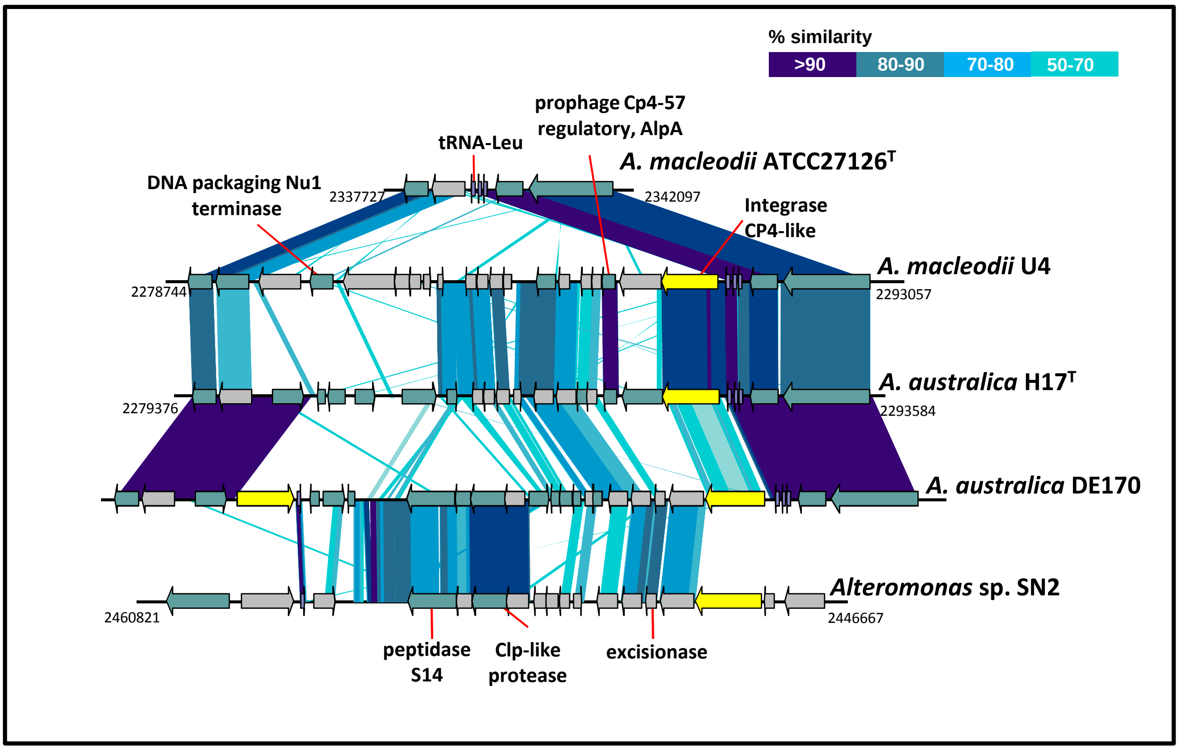

Additional Figure S7.- Putative Prophage CP4-57-like found in both *A. australica* and comparison to the similar prophages found *A. macleodii* U4, *Alteromonas* sp. SN2 and *A. macleodii* ATCC27126^{T}

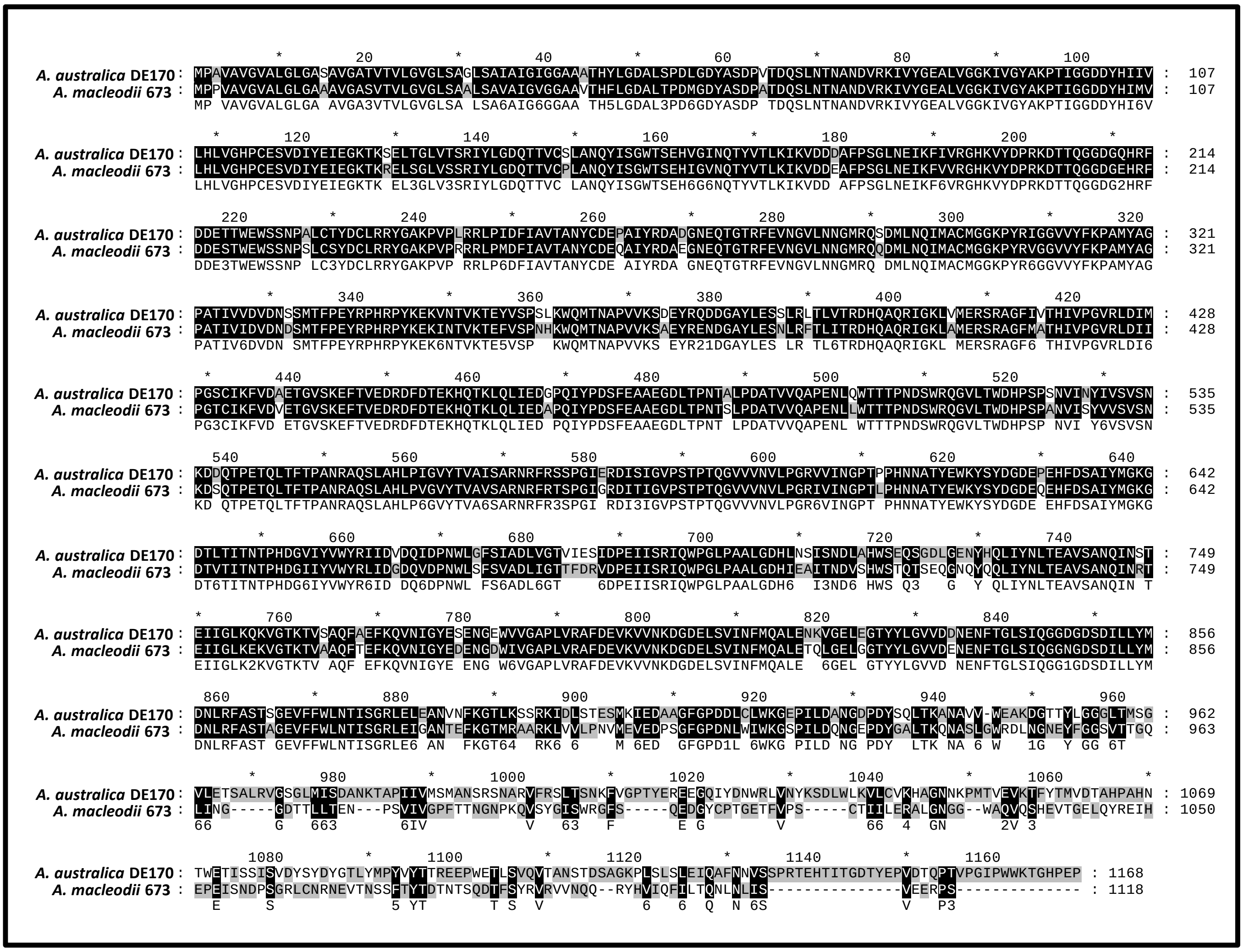

Additional Figure S8.- Alignment of the tail fiber protein from Mu-like prophages inserted in the *A. australica* DE170 and *A. macleodii* 673 genomes

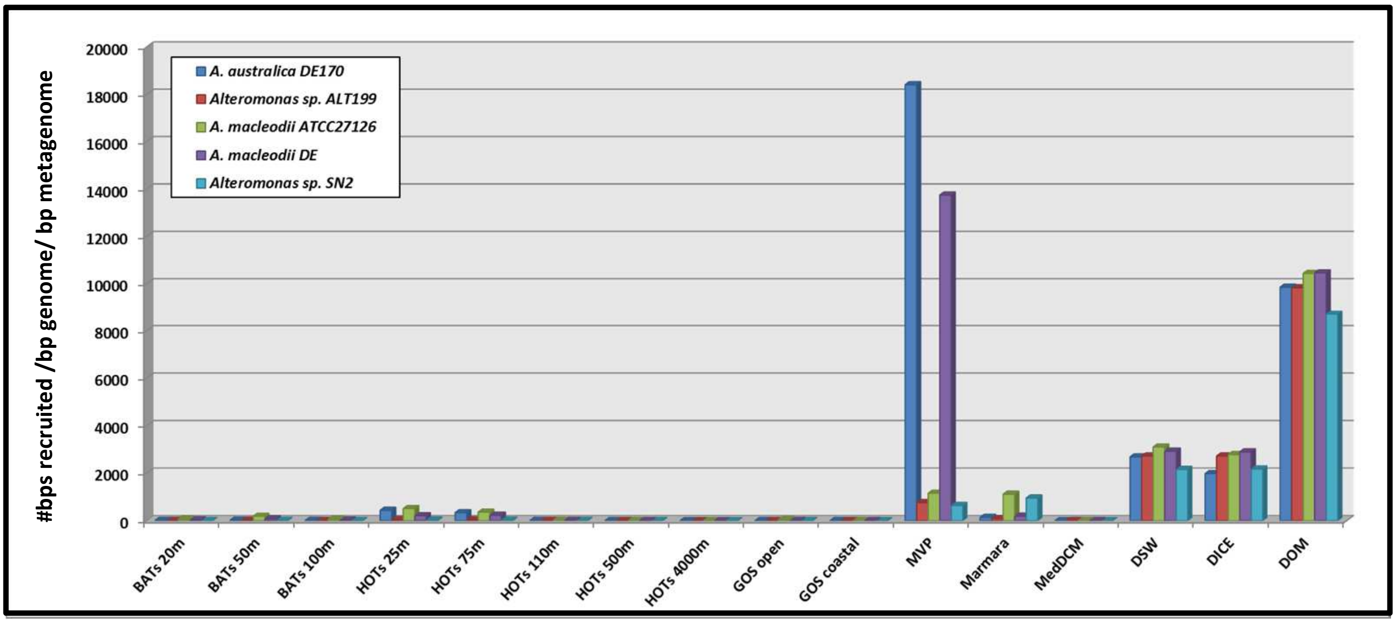

Additonal Figure 9. *Alteromonas* species genomes relative recruitment of metagenomic reads at 98% identity and 90% coverage from some marine reference metagenomes. [BATs (Bermuda Atlantic Time Series) [56], DICE (Dauphin Island Cubitainer Experiment) [60], DOM (Dissolved Organic Matter) [7], DSW (Deep Sea Water) [6], GOS (Global Ocean Survey) [58], HOTs (Hawaii Ocean Time Series) [57, 82], Marmara [83], MedDCM (Mediterranean Deep Chlorophyll Maximum) [84] and MVP (Matapan-Vavilov Deep) [59]].

Table S1. Features of the reference genomes

Organism/Name	BioSample	BioProject	Assembly	Size (Mb)	GC%	RefSeq	Status
***Alteromonas macleodii* ATCC 27126**	SAMN02603229	PRJNA55253	GCA_000172635.2	4.65385	44.7	NC_018632.1	finished
***Alteromonas macleodii* AltDE1**	SAMN02604119	PRJNA179068	GCA_000310085.1	4.94713	44.7	NC_019393.1	finished
***Alteromonas macleodii* str. 'Aegean Sea MED64'**	SAMN02604122	PRJNA231689	GCA_000439495.1	4.39754	44.8	NC_023045.1	finished
***Alteromonas macleodii* str. 'Balearic Sea AD45'**	SAMN02604120	PRJNA176366	GCA_000300175.1	4.67488	44.7	NC_018679.1	finished
***Alteromonas macleodii* str. 'Black Sea 11'**	SAMN02604118	PRJNA176365	GCA_000299995.1	4.48051	44.6	NC_018692.1	finished
***Alteromonas macleodii* str. 'Deep ecotype'**	SAMN02603915	PRJNA58251	GCA_000020585.3	4.48094	44.9	NC_011138.3	finished
***Alteromonas macleodii* str. 'English Channel 615'**	SAMN02604124	PRJNA210781	GCA_000439475.1	4.58235	44.9	NC_021716.1	finished
***Alteromonas macleodii* str. 'English Channel 673'**	SAMN02604121	PRJNA176367	GCA_000299955.1	4.60178	44.8	NC_018678.1	finished
***Alteromonas macleodii* str. 'Ionian Sea U4'**	SAMN02604123	PRJNA210780	GCA_000439515.1	4.62458	44.7	NC_021710.1	finished
***Alteromonas macleodii* str. 'Ionian Sea U7'**	SAMN02604128	PRJNA210785	GCA_000439535.1	4.44294	44.8	NC_021717.1	finished
***Alteromonas macleodii* str. 'Ionian Sea U8'**	SAMN02604125	PRJNA210782	GCA_000439555.1	4.39504	44.9	NC_021712.1	finished
***Alteromonas macleodii* str. 'Ionian Sea UM4b'**	SAMN02604127	PRJNA210784	GCA_000439595.1	4.43877	44.9	NC_021714.1	finished
***Alteromonas macleodii* str. 'Ionian Sea UM7'**	SAMN02604126	PRJNA210783	GCA_000439575.1	4.93166	44.7	NC_021713.1	finished
***Alteromonas* sp. SN2**	SAMN02603134	PRJNA67349	GCA_000213655.1	4.97215	43.5	NC_015554.1	finished
***Alteromonas* sp. ALT199**	-	PRJNA190838	GCA_000597705.1	4.63567	43.7	JFBK01000001	draft

Additional Material and Methods

Transcriptomic analysis. RNA sequencing was used to analyze transcriptional changes between both the growth conditions examined (e.g., temperature and medium) as well as differences between the two A. macleodii strains DE and DE1 within a given condition. The conditions assayed included two temperatures (13 and 25°C), which represent normal temperature extremes in the Mediterranean (winter and summer respectively), and two growth media, a commonly used laboratory medium (marine agar or nutrient-rich medium, RM) and minimal medium with glucose (MMG). Another condition assayed was starvation (STR), in which cells were deprived of any carbon source for 48 hours. cDNA of these four growth conditions in AltDE and AltDE1 were sequenced and mapped to the genomes.
